# USP1-trapping lesions as a source of DNA replication stress and genomic instability

**DOI:** 10.1038/s41467-022-29369-3

**Published:** 2022-04-01

**Authors:** Kate E. Coleman, Yandong Yin, Sarah Kit Leng Lui, Sarah Keegan, David Fenyo, Duncan J. Smith, Eli Rothenberg, Tony T. Huang

**Affiliations:** 1 Department of Biochemistry & Molecular Pharmacology, New York University School of Medicine, New York, NY USA; 2 Institute for Systems Genetics, New York University School of Medicine, New York, NY USA; 3 Department of Biology, New York University, New York, NY USA; 4 Present Address: Institute of Chemical Biology, Shenzhen Bay Laboratory, School of Chemical Biology and Biotechnology, Peking University Shenzhen Graduate School, Shenzhen, China

**Keywords:** Post-translational modifications, DNA replication

## Abstract

The deubiquitinase USP1 is a critical regulator of genome integrity through the deubiquitylation of Fanconi Anemia proteins and the DNA replication processivity factor, proliferating cell nuclear antigen (PCNA). Uniquely, following UV irradiation, USP1 self-inactivates through autocleavage, which enables its own degradation and in turn, upregulates PCNA monoubiquitylation. However, the functional role for this autocleavage event during physiological conditions remains elusive. Herein, we discover that cells harboring an autocleavage-defective USP1 mutant, while still able to robustly deubiquitylate PCNA, experience more replication fork-stalling and premature fork termination events. Using super-resolution microscopy and live-cell single-molecule tracking, we show that these defects are related to the inability of this USP1 mutant to be properly recycled from sites of active DNA synthesis, resulting in replication-associated lesions. Furthermore, we find that the removal of USP1 molecules from DNA is facilitated by the DNA-dependent metalloprotease Spartan to counteract the cytotoxicity caused by “USP1-trapping”. We propose a utility of USP1 inhibitors in cancer therapy based on their ability to induce USP1-trapping lesions and consequent replication stress and genomic instability in cancer cells, similar to how non-covalent DNA-protein crosslinks cause cytotoxicity by imposing steric hindrances upon proteins involved in DNA transactions.

## Abstract (web summary)

Here the authors provide mechanistic insights into how auto-cleavage of the USP1 deubiquitinase regulates DNA replication and genome stability. Implications for the targeting of USP1 activity via protein-DNA trapping in cancer therapy are discussed.

## Introduction

Reversible protein ubiquitylation is critical for the maintenance of genome integrity, both in unperturbed conditions and in response to replication fork-stalling events induced by genotoxic agents^1,2^. Maintenance of appropriate cellular protein ubiquitylation levels involves tightly coordinated ubiquitin conjugation to substrates by E3 ubiquitin ligases, and ubiquitin removal via deubiquitinases (DUBs)^3,4^. Over 100 DUBs have been identified in the human genome belonging to six subfamilies, which remove ubiquitin modifications by cleaving the isopeptide bond between ubiquitin and the modified protein^5,6^. As DUB activity is commonly deregulated in a wide variety of human cancers^7^, there has been growing interest in targeting DUBs therapeutically to modulate critical cellular processes such as DNA replication and repair in cancer cells.

The ubiquitin-specific protease 1 (USP1), along with its catalytic co-factor, UAF1 (WDR48), is a DUB with critical functions in the maintenance of genome integrity through the deubiquitylation of the proliferating cell nuclear antigen (PCNA), and other cellular substrates^8,9^. PCNA monoubiquitylation at Lys164 is particularly important in facilitating the rescue of stalled replication forks by promoting translesion synthesis (TLS), a DNA damage tolerance (DDT) pathway that employs a class of low-fidelity Y-family TLS polymerases to directly bypass many lesions and distortions in DNA^10–12^. When replication forks encounter DNA lesions, the E3 ubiquitin ligase RAD18 monoubiquitylates PCNA, which in turn promotes the recruitment of TLS polymerases (such as Pol Eta, Kappa, Iota, or Rev1) via their ubiquitin-binding domains (UBDs) and weak PCNA-interacting peptide (PIP) box interactions^13–15^. Although this pathway allows for the resumption of DNA replication during DNA damage or replication stress, the use of low fidelity TLS polymerases poses a risk for the increased incorporation of mutations into DNA. Thus, by reversing PCNA monoubiquitylation, USP1 helps to limit the unscheduled deployment of error-prone TLS polymerases to preserve genome integrity^8^. Accordingly, dysregulation of USP1 activity can contribute to a variety of DNA replication defects, including aberrant PCNA monoubiquitylation and recruitment of the TLS polymerase Pol Kappa (PolK), increased mutational frequency, decreased replication fork speed, hypersensitivity to crosslinking agents, and micronuclei formation^8,16–18^. Recent studies have even suggested that USP1 has DNA-binding activity and that USP1-WDR48 complexes localize to stalled replication forks^19,20^. However, it is unclear how USP1 is regulated to serve a functional role during DNA replication.

The protein level or stability of USP1 is regulated through different mechanisms (reviewed in^21,22^). One of these mechanisms is through DUB autocleavage (self-cleavage through its own protease activity), which is best-observed upon exposure to UV irradiation, causing a rapid degradation of USP1 protein^8^. USP1 autocleavage occurs immediately proximal to an internal conserved ubiquitin-like diglycine (Gly-Gly) motif at residues 670–671 and is dependent on the catalytic activity of USP1^8^. The DNA damage-induced autocleavage of USP1 coincides with the upregulation of PCNA monoubiquitylation levels and the proteasome-dependent degradation of USP1 by two separate pathways^8^: The N-terminal autocleavage product contains a C-end degron that is recognized and eliminated through DesCEND, a mechanism by which the CRL2 ubiquitin ligase uses interchangeable substrate receptors to target proteins with unusual C-termini (in this case, the C-terminal Gly-Gly of USP1)^23^, and the smaller C-terminal autocleavage product is targeted for degradation via the Arg/N-end rule pathway through deamidation of its destabilizing N-terminal Gln residue^24^. However, it is unknown whether USP1 autocleavage occurs solely for degradation purposes or whether it serves additional roles that affect cellular processes.

Here, we address this knowledge gap by elucidating USP1 spatiotemporal dynamics at replication forks and its associated cellular phenotypes. To determine the functional role of USP1 autocleavage, we employed CRISPR-Cas9 tools to generate bi-allelic knock-in mutations of USP1 in human cells and set out to measure endogenous effects of defective USP1 autocleavage and catalytic activity on DNA replication dynamics in unperturbed conditions. Surprisingly, we discovered that cells harboring an autocleavage mutant of USP1 (USP1-GG/AA) are significantly more impaired in their ability to maintain proper replication fork elongation in comparison to wildtype and USP1 knock-out (KO) cells. To better understand the mechanism behind this replication fork impairment, we applied single-molecule localization microscopy (SMLM) and live-cell single-molecule tracking (SMT) to show increased retention of USP1 molecules (USP1-trapping) at sites of nascent DNA synthesis in autocleavage-defective mutants. Interestingly, wildtype cells treated with a USP1 inhibitor (ML323), as well as cells harboring catalytically-inactive USP1 (USP1-C90S) accumulate USP1-trapping lesions on DNA, which can be partially alleviated by the DNA-dependent metalloprotease Spartan (SPRTN). Furthermore, we found that ubiquitylated PCNA enables the recruitment of SPRTN to remove these trapped DNA-protein complexes in cells to ensure genomic stability. Overall, our findings elucidate a mechanism for steady-state USP1 autocleavage in maintaining replication fork integrity and, thus, inform new therapeutic strategies to exploit the cellular intolerance of protein-DNA trapping lesions for cancer treatments.

## Results

### USP1 autocleavage is required for normal DNA replication fork elongation

**Fig. 1:**
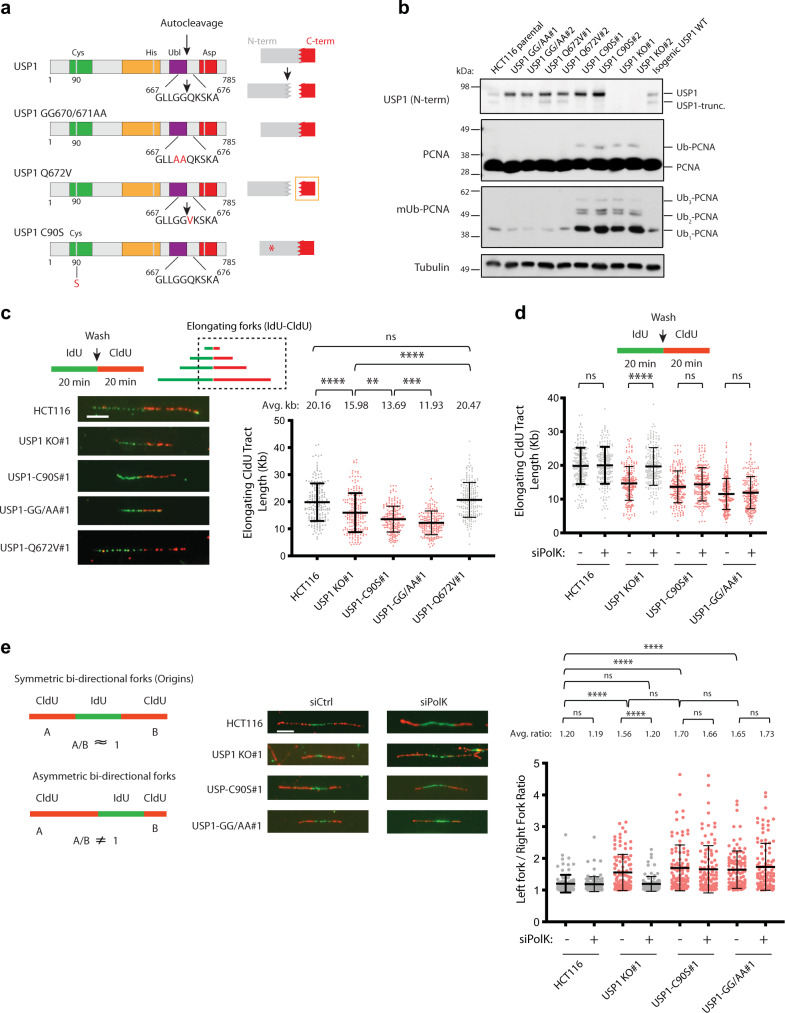
Impaired USP1 autocleavage contributes to reduced replication fork speed and fork stalling. **a** Schematic representations of USP1 mutants utilized in this study. **b** Whole-cell lysates of HCT116 USP1 mutant cell lines were analyzed by immunoblotting with the indicated antibodies. For this figure and all subsequent figures, tubulin serves as a loading control. Iso. USP1 represents an isogenic wild-type control derived following clonal selection of USP1 mutant cell lines. **c** (left panel) Representative images of elongating DNA fiber tracts from HCT116 parental cell line and USP1 knock-in clones. Scale bar = 5 μm. (right panel) Scatter plots show CldU tract length measurements from elongating forks for three independent experiments (*n* = 200 elongating forks) with mean and −/+ SD indicated. *p* values were calculated using the Mann–Whitney rank-sum t-test (ns = no significance, **p < 0.01, ***p < 0.001, ****p < 0.0001, two-tailed). **d** HCT116 parental and USP1 mutant cell lines were treated with either control or Polκ siRNA for 72 h, and subsequently pulse-labeled with IdU and then CldU for 20 min. CldU tract lengths were measured in each sample to assess relative speed of elongating forks. Data are plotted for 3 independent experiments (*n* = 200 elongating forks) with mean and −/+ SD indicated and *p* values were calculated using the Mann–Whitney rank-sum t-test (ns = no significance, **** = *p* < 0.0001, two tailed). **e** Schematic for assessment of symmetrical vs. asymmetrical fork progression in HCT116 cell lines transfected with either control or Polκ siRNAs, as in (**d**). Representative images show bidirectional forks emanating from a single origin for the indicated cell lines. Scale bar = 5 μm. Scatter plots depict ratios of CldU tract length measurements from leftward- versus rightward-moving forks emanating from the same origin of replication (*n* = 100 bi-directional forks (origins)), with mean and −/+ SD indicated. p values were calculated using the Mann–Whitney rank-sum t-test (ns = no significance, ****p < 0.0001, two-tailed).

To study the endogenous effects of impaired USP1 autocleavage on DNA replication dynamics, we generated different HCT116 cell lines harboring biallelic knock-in mutations of USP1 using CRISPR/Cas9 gene-editing methods (see schematics, Fig. 1a). These USP1 mutant cell lines include: (1) an autocleavage-deficient mutant with alanine substitutions at residues 670–671 (GG/AA mutant); (2) a Q672V mutant, which produces a stable C-terminal autocleavage product that cannot be properly recognized for degradation by the Arg/N-end rule pathway; (3) a catalytically-inactive mutant (C90S); and (4) USP1 knockout (KO) clones. All USP1 mutations and gene deletions were validated using Next Generation Sequencing (NGS) analysis (Applied Stem Cell Technologies). To validate the functional effects of the endogenous USP1 mutants in the different HCT116 cell lines, we showed by Western blot that the USP1-WT and USP1-Q672V cells produced steady-state autocleaved USP1 (indicated by the presence of a truncated USP1 N-terminal fragment, USP1-trunc.), while the autocleaved products were absent in both the USP1-GG/AA and USP1-C90S mutant cell lines (Fig. 1b). Using the DUB activity probe, ubiquitin vinylsulfone (HA-Ub-VS), we showed that both the endogenous USP1-GG/AA and USP1-Q672V mutant proteins are catalytically competent (reactive with the HA-Ub-VS probe to form a slower-migrating covalent protein complex), but not the USP1-C90S mutant (Supplementary Fig. 1a). We also used cycloheximide treatment to measure the relative protein turnover of the USP1 mutants and observed an increase in protein stability of the USP1-GG/AA, Q672V, and C90S endogenous proteins in comparison to USP1-WT; this is in line with our previous observations (Supplementary Fig. 1b)^8,24^. As an additional indicator of endogenous USP1 activity in each of these cell lines, we probed Western blots with antibodies against total PCNA and monoubiquitylated (mUb)-PCNA to monitor cellular PCNA ubiquitylation levels. As predicted, increased levels of ubiquitylated PCNA (Ub-PCNA) were observed in both the USP1-C90S and USP1 KO cell lines, whereas decreased PCNA ubiquitylation levels were detected in USP1-GG/AA and USP1-Q672V cells in comparison to the parental HCT116 cell line (Fig. 1b). These results are in agreement with our prior studies showing that ectopically-expressed USP1-GG/AA and USP1-Q672V mutants are more stable than the wildtype counterpart, while still retaining DUB activity^24,25^.

In our previous study, we demonstrated that USP1 plays an important role in the maintenance of genomic stability by preventing aberrant PCNA ubiquitylation and TLS polymerase kappa (PolK) recruitment^18^. Here, we used single-molecule DNA fiber assay to compare and contrast how different endogenous USP1 mutants could impact replication fork elongation rates. HCT116 cells were pulse-labeled sequentially with iododeoxyuridine (IdU) and chlorodeoxyuridine (CldU) analogs to determine the polarity of elongating replication forks, and tract lengths of the second label were measured for assessment of fork speed (see schematics, Fig. 1c). Consistent with our previous findings^18^, we observed a reduction in fork speed with the USP1 KO cell lines in comparison to WT parental cells (Fig. 1c and Supplementary Fig. 1c). We also observed a more severe fork speed effect in the USP1-C90S cells in comparison to the USP1 KO cell lines, indicative of a dominant-negative effect of the catalytic mutant (Fig. 1c). Surprisingly, despite having robust USP1 DUB activity, the USP1-GG/AA mutant cells produced the strongest reduction in replication fork speed (Fig. 1c). This effect is likely due to the inability of the GG/AA mutant to be autocleaved rather than its increased protein stability, as the autocleavage-proficient and yet highly-stabilized USP1-Q672V mutant showed relatively normal elongating fork speed (Fig. 1c). Replication fork speed was also measured in alternative clones for each of the USP1 mutant cell lines with similar differences (Supplementary Fig. 1c). Taken together, these results suggest that in addition to USP1 DUB activity, USP1 autocleavage acts as an independent modulator of proper replication fork elongation in unperturbed conditions.

### Impaired USP1 autocleavage causes increased replication fork-stalling in a Polκ-independent manner

We previously showed that co-depletion of the TLS polymerase PolK rescues the reduction in replication fork speed observed in USP1-depleted cells, suggesting that over-engagement of PolK with Ub-PCNA impedes fork progression^18^. Therefore, we sought to determine whether the depletion of PolK could similarly rescue the reduction in fork speed observed in the USP1 autocleavage-deficient cell lines. In agreement with our previous results, siRNA-mediated knockdown of PolK in the USP1 KO cell line resulted in restoration of elongating fork speed to WT levels (Fig. 1d and Supplementary Fig. 1d). However, depletion of PolK in the USP1-C90S and USP1-GG/AA mutant background did not rescue the reduced fork speed observed in the cells, indicating that the USP1-C90S and USP1-GG/AA mutants inhibited fork progression independently of PolK (Fig. 1d and Supplementary Fig. 1d). Overall, these results indicate that the reduced replication fork speed observed in both the USP1-C90S and USP1-GG/AA mutant cell lines are not dependent on aberrant PolK usage.

Next, we wanted to determine whether the decreased fork speed observed in the different USP1 mutant cell lines also led to increased frequency of replication fork-stalling events. Taking advantage of the dual-labeling strategy used in our DNA fiber assay, we analyzed fork-stalling events by measuring the fork lengths of two bidirectional forks emanating from the same origin of replication, as previously described^25,26^. In unperturbed conditions, it is predicted that the bi-directional forks will generally travel at the same speed (similar fork-lengths), resulting in the ratio of their tract lengths being close to 1 (as illustrated in Fig. 1e). On the other hand, fork-stalling can be visualized as asymmetric fork progression, with bi-directional forks emanating from the same origin at different rates (ratio ≠ 1) (Fig. 1e). We observed a significantly higher frequency of asymmetric forks in the USP1 KO cells than in the parental HCT116 cell line, consistent with the increased occurrence of fork-stalling in this background (Fig. 1e and Supplementary Fig. 1d). Notably, fork-stalling in the USP1 KO cell line was alleviated by PolK depletion, suggesting that the aberrant retention of PolK at the fork is largely responsible for the increased incidence of fork-stalling in USP1 KO cells (Fig. 1e and Supplementary Fig. 1e). Importantly, the USP1-C90S and USP1-GG/AA mutant cell lines also produced much higher frequencies of asymmetric fork progression than WT cells, which were not significantly affected by siRNA-mediated PolK depletion (Fig. 1e and Supplementary Fig. 1e). These data support the notion that impaired USP1 autocleavage also causes an increased frequency of fork-stalling, indicative of endogenous replication stress, but in a manner that is independent of PolK.

### Defective USP1 autocleavage results in increased levels of replication-associated DNA damage

Since increased fork-stalling can contribute to replication stress and DNA damage, we next investigated whether DNA damage checkpoints were elevated in unperturbed conditions for USP1 KO, USP1-C90S, and USP1-GG/AA cell lines. In agreement with previous results^18^, neither USP1 KO nor endogenous expression of USP1-C90S and USP1-GG/AA mutants caused any increase in checkpoint signaling above basal level, as measured by Chk1 phosphorylation (Supplementary Fig. 2a). In response to higher levels of fork-stalling events, the increased efficiency of ‘dormant’ origin firing can help compensate for reduced fork speed to ensure timely genome replication in mammalian cells^27,28^. We analyzed the frequency of origin firing of each of the USP1 mutant cell lines by counting the number of origin initiation events (both bi-directional red-green-red tracts and red only tracks) over the total number of replication structures (Supplementary Fig. 2b). Consistent with published results^29^, an increase in origin firing frequency was measured in the USP1 KO cell lines in comparison to wild-type cells (Supplementary Fig. 2b). Notably, the autocleavage-deficient cell lines (both USP1-GG/AA and USP1-C90S cells) also contained increased number of origins in comparison to both the USP1-WT and USP1-Q672V mutant cell lines to account for decreased fork speed. Thus, we found that origin firing levels are increased in the USP1 autocleavage mutant cell lines to counter-balance for the overall reduction in replication fork speed. Accordingly, flow cytometry analysis of DNA synthesis (marked by EdU) versus DNA content (DAPI staining) revealed that the proportion of cells in S phase was similar between WT cells and each of the USP1 mutant cell lines, suggesting that S phase progression was not substantially impacted (Supplementary Fig. 2c).

In addition, low levels of unresolved replication stress due to under-replication of DNA in S phase can also result in the generation of DNA lesions, which are then transmitted to daughter cells in the subsequent G1 phase in 53bp1 nuclear bodies^30,31^. To assess this form of replication-associated DNA damage, we analyzed the USP1 WT and mutant cell lines for the presence of 53BP1 nuclear bodies in Cyclin A-negative G1 cells (Supplementary Fig. 2d). Interestingly, increased percentages of G1 cells with 53BP1 nuclear bodies were detected in USP1 KO and autocleavage-defective USP1-GG/AA and USP1-C90S cells in comparison to WT cells, suggestive of under-replicated DNA in the previous S phase (Supplementary Fig. 2d). On the other hand, the USP1-Q672V cell line resembled WT cells with respect to the formation of 53BP1 nuclear bodies in G1 (Supplementary Fig. 2d). Thus, we conclude that the replication defects observed in the USP1 KO, GG/AA, and C90S cell lines contribute to increased levels of endogenous replication stress and genome instability.

### Impaired USP1 autocleavage causes delocalized replication origin initiation and termination in transcriptionally-active long genes

**Fig. 2:**
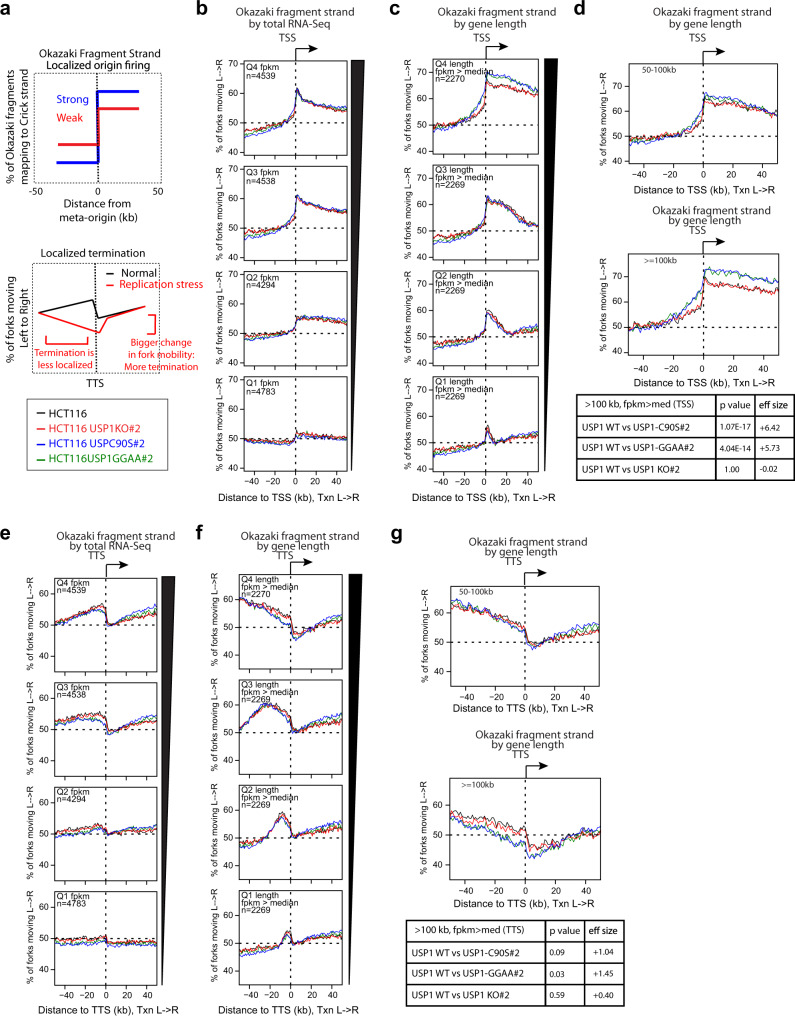
Delocalized patterns of replication initiation and termination within long genes in USP1 autocleavage mutant cell lines. **a** (upper panel) Schematic showing anticipated Okazaki fragment distributions around replication origins with strong (blue) versus weak (red) localized firing efficiencies. (lower panel) Schematic representation of Okazaki fragment distributions arising from replication termination at TTS under normal conditions (black) versus delocalized termination under replication stress (red). **b** Percentage of replication forks moving left to right around TSS binned by total RNA-seq read depth quartile (FPKM), (from ref. ^64^) for the indicated cell lines. **c** Percentage of replication forks moving left to right around TSS of actively transcribed genes (FPKM>median), binned by gene length according to quartiles for transcribed genes. **d** Same analysis as in (**c**), binned for long genes of 50–100 kb (upper panel) or >100 kb (lower panel). **e** Percentage of replication forks moving left to right around TTS binned by RNA-seq read depth quartile (from ref. ^64^) for the indicated cell lines. **f** Percentage of replication forks moving left to right around TTS of actively transcribed genes (FPKM>median), binned by gene length according to quartiles for transcribed genes. **g** Same analysis as in (**f**), binned for long genes of 50–100 kb (upper panel) or >100 kb (lower panel). *P* values displayed in tables were calculated using the Kruskal–Wallis test, using the regions ±1–10 kb around TSS and TTS of long genes (>100 kb). Effect sizes were calculated using the same regions. The values were calculated comparing the USP1 mutant cell lines against the USP1 wild-type cell line.

Next, we wanted to determine how impairment of USP1 autocleavage could be altering genome-wide DNA replication dynamics in unperturbed conditions. In our previous study, we utilized whole-genome Okazaki fragment sequencing (Ok-seq) to define and quantitatively compare sites of replication initiation and termination in untransformed human cells^32^. This method has proven to be useful in detecting genome-wide changes in replication fork directionality, which can be indicative of how replication stress modulates replication origin firing efficiency, replication elongation, and termination in both *S. cerevisiae* and mammalian systems^32–35^. For instance, usage of a highly efficient replication origin will manifest as an increase in the percentage of sequenced Okazaki fragments mapping to the Crick strand moving from the arbitrary left fork to right (L→R), whereas a site of replication termination generates a transition in Okazaki fragment strand bias opposite to that observed at replication origins (observed as a decreased proportion of forks moving from L→R) (see schematics, Fig. 2a)^32,34,35^. Using Ok-seq analysis, we previously found that replication forks are preferentially established at transcription start sites (TSS) of genes occupied by RNA Pol II (active genes) and terminate in regions localized to the transcription termination site (TTS) of transcribed genes, thus ensuring co-directionality of replication forks with the transcription machinery to minimize the hazards of transcription-replication conflicts (TRCs)^32^. On the other hand, under conditions of replication stress induced by hydroxyurea (HU), replication origin efficiency increases not only near TSS of active genes, but also downstream of genes, reorienting replication relative to gene 3′ ends, while termination events become more delocalized (Fig. 2a)^32^.

Given our observations of increased fork-stalling in the HCT116 cell lines defective for USP1 autocleavage, we used Ok-seq analysis to determine whether there are any differences in origin firing efficiency or localization at TSS, as well as the extent and localization of termination at TTS. In brief, we pulse-labeled nascent DNA from WT and USP1 mutant cell lines with the nucleotide analog 5-Ethynyl-2′-deoxyuridine (EdU), isolated Okazaki fragments using a 5–30% linear sucrose gradient, purified EdU-labeled and biotin-conjugated Okazaki fragments using streptavidin resin, and prepared libraries of Okazaki fragments ligated to Illumina adapters for paired-end sequencing^32,35^. Fractions from the linear sucrose gradient were analyzed on an alkaline gel to ensure that only fragments <200 bp were captured for sequencing analysis, and prepared Okazaki fragment libraries were analyzed using Agilent Tapestation to ensure sufficient yield and purity prior to Illumina sequencing (Supplementary Fig. 3a–c). We then calculated the percentages of forks moving from left to right by performing meta-analysis of Okazaki fragment distributions around specific genomic loci (TSS or TTS).

To determine the efficiency and localization of replication origin firing, we calculated Okazaki fragment strand bias within a 50 kb window surrounding all annotated TSS in the human genome. Genes were separated into quartiles based on RNA-seq read density from total RNA (fragments per kilobase of transcript per million mapped reads, FPKM) (Fig. 2b) or length of transcribed genes (Fig. 2c). Consistent with our previous study, we observed that in all cell lines analyzed, the TSS of genes with higher FPKM showed increased change in Okazaki fragment strand bias compared to TSS of weakly or non-transcribed genes (Fig. 2b). Likewise, gene length was positively correlated with origin efficiency (increased percentage of forks moving co-directionally through the gene body) (Fig. 2c). In evaluating the different HCT116 cell lines, we noted a significant increase in Okazaki fragment strand bias correlating with gene length in the autocleavage-deficient USP1-GG/AA and USP1-C90S cell lines in comparison to WT cells (Fig. 2c). This was particularly evident at the TSS of genes greater than 100 kb in length, indicating elevated origin activity at TSS of the longest genes (Fig. 2d). Additionally, the change in Okazaki fragment strand bias, indicative of origin firing, occurs over a broader range in cell lines with defective USP1 autocleavage, suggesting that origin firing is more delocalized in these strains as opposed to occurring in the immediate vicinity of TSS (Fig. 2d). By contrast, the USP1 KO cell lines resembled WT in terms of Okazaki fragment strand bias at TSS of long genes. A likely explanation for this is that the fork slowdown/stalling is more stochastic in the USP1 KO cells due to the pleiotropic effects of aberrant PolK usage that may not exclusively occur in the vicinity of TSS, thus making it more difficult to capture by the Ok-seq technique.

We then quantified replication termination by calculating Okazaki fragment strand bias within a 50 kb window relative to all annotated polyadenylation sites (TTS). In agreement with our previous results, increased FPKM correlated with a sharp reduction in the percentage of replication forks moving from left to right at TTS (Fig. 2e); although replication termination can occur throughout the genome, replication fork convergence is localized preferentially at the 3′ end of transcribed genes. The sharp decrease in rightward-moving replication forks at TTS, indicative of replication fork termination, was also correlated with gene length in all cell lines, with a trend towards more intragenic termination in the longest genes (Fig. 2f). Interestingly, in our analysis of long genes from Okseq library preps of USP1-GG/AA and USP1-C90S cells, while replication termination was still elevated around TTS, we observed a reduction in replication forks moving co-directionally through gene bodies in the window of −20 kb before TTS, indicating more intragenic termination. Moreover, a greater change in fork mobility was observed in the 20 kb region following TTS, suggesting an overall increase in termination events (Fig. 2f). These effects were especially prominent around the TTS of very long genes (Fig. 2g). Similar patterns of delocalized replication initiation and termination around long genes were observed in different clones of the USP1-GG/AA and USP1-C90S cell lines (Supplementary Fig. 4). Taken together, these results indicate that the impairment of USP1 autocleavage, which causes increased fork-stalling, leads to premature and delocalized fork termination around the TTS of transcribed long genes.

### Single-molecule imaging analysis provides evidence for USP1 “trapping” at sites of nascent DNA synthesis

**Fig. 3:**
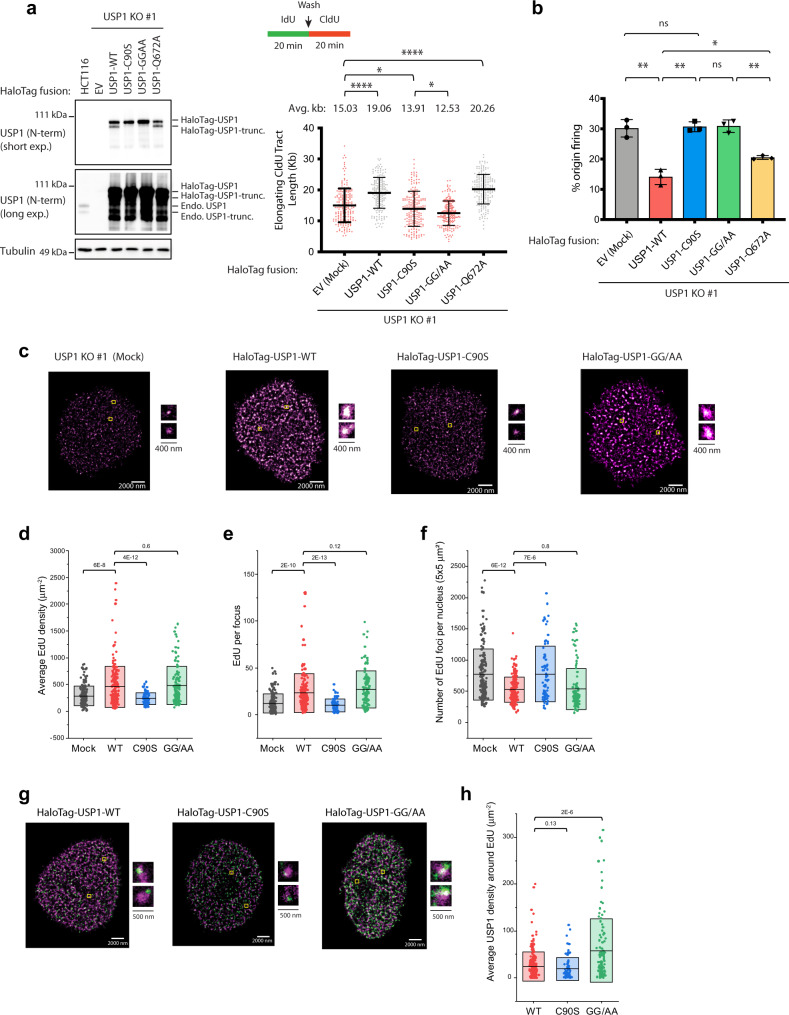
USP1 autocleavage mutant colocalizes strongly with sites of nascent DNA synthesis. **a** HCT116 USP1 KO cells complemented with the indicated HaloTag-USP1 fusion constructs were either subjected to immunoblotting analysis (left panel) or DNA fiber analysis of elongating fork speed (right panel). Data are graphed with mean and −/+ SD for 3 independent experiments (*n* = 200 elongating forks), and p values were calculated using the Mann–Whitney rank-sum t-test (*p < 0.05, ****p < 0.0001, two-tailed). **b** Cells treated as in (**a**) were analyzed using the DNA fiber assay for % origin firing. See Supplementary Fig. 2b for criteria for scoring of replication origins. Data for % origin firing are represented by mean and −/+ SD for three independent experiments (*n* = 200 events) and *p*-values were calculated using t-test with Welch’s correction (ns = no significance, *p < 0.05, **p < 0.01, two-tailed). **c** Untreated HCT116 USP1 KO cells expressing the indicated HaloTag-USP1 fusion constructs were labeled with 10 μM EdU and 20 nM JF549 HaloTag ligand for 15 min prior to CSK extraction of soluble proteins and fixation. **d**–**f** Representative super-resolution images with EdU signal in magenta are shown. Quantifications of average EdU density per nucleus (**d**), EdU intensity per focus (**e**), and number of EdU foci per nucleus (**f**) are plotted for each sample, based on at least two independent experiments (mean −/+ std, *n* = 186, 196, 76, 113 nuclei for Mock, USP1-WT, USP1-C90S, and USP1-GG/AA, respectively, *p*-values were calculated using the student’s t-test; mean and std are represented as the middle bar and the box, respectively). **g** Representative super-resolution images showing distribution of both the HaloTag-USP1 fusions (green) and EdU-labeled DNA. **h** Measurements of average USP1 density around EdU are shown, based on at least two independent experiments (mean −/+ std, *n* = 188, 71, 113 nuclei for USP1-WT, USP1-C90S, and USP1-GG/AA, respectively, *p*-values were calculated using the student’s t-test; mean and std are represented as the middle bar and the box, respectively).

Considering our findings of increased fork-stalling in cell lines with impaired USP1 autocleavage (USP1-C90S and USP1-GG/AA mutants), we hypothesized that increased retention of USP1 at the fork could be impeding fork progression. To determine this, we sought to quantify the specific level of association of individual USP1 molecules (WT and mutant proteins) at replication forks by utilizing the multi-color SMLM approach. The increased sensitivity and spatial resolution of SMLM provides nanometer scale features of protein localization at replication forks, which cannot be resolved via diffraction-limited fluorescence microscopy^36–38^. To our knowledge, the use of conventional indirect immunofluorescence microscopy techniques had previously failed to detect USP1 at DNA damage/repair sites. To directly analyze the distribution and association of individual USP1 molecules with sites of nascent DNA synthesis, we generated N-terminal HaloTag fusion constructs of WT and each of the USP1 mutants. This enabled us to specifically label HaloTag-USP1 molecules via HaloTag ligand conjugated to JF549, whereas nascent DNA was detected by fluorescent-labeling (with AF647) of the nucleotide analog 5-Ethynyl-2′-deoxyuridine (EdU) that was incorporated for 15 min (see “Methods”). By transiently expressing the HaloTag-USP1 constructs in USP1 KO cells, we were able to show that WT (and USP1-Q672A), but not the autocleavage-deficient HaloTag-USP1-C90S and HaloTag-USP1-GG/AA fusion constructs, were able to functionally complement the fork elongation defect by DNA fiber assay (Fig. 3a). Moreover, increased origin firing frequency coincided with the reduced fork speed observed in cells complemented with the HaloTag-USP1-C90S and HaloTag-USP1-GG/AA fusion constructs (Fig. 3b). These data are in agreement with our results from the HCT116 USP1 knock-in mutant cell lines (Fig. 1c and Supplementary Fig. 2b).

To gain better insight into the distribution of the SMLM-resolved replication sites in cells complemented with the HaloTag-USP1 fusions, we pulse-labeled cells with EdU and measured differential EdU fluorescence intensities as derived from the auto-correlation analysis^36,38,39^ according to the following parameters: Average EdU density per nucleus (Fig. 3d), average EdU counts per replication site (Fig. 3e), and number of EdU foci per nucleus (this corresponds to the average number of active forks per area) (Fig. 3f) (see representative images of EdU signal for each sample in Fig. 3c). Strikingly, in comparison to WT-complemented cells, we observed a marked reduction in both average EdU intensity per nucleus as well as EdU counts per focus in USP1 KO cells that were either mock-treated or complemented with the HaloTag-USP1-C90S fusion construct, indicating a decreased overall amount of DNA synthesis (Fig. 3d, e). This was accompanied by an increased number of EdU foci per nucleus, which likely signifies an increase of replication factories/events to help compensate for the overall reduction of DNA synthesis from each replication fork (Fig. 3f). Surprisingly, USP1 KO cells complemented with the HaloTag-USP1-GG/AA mutant resembled WT-complemented cells across all measurements, indicating wild-type levels of nascent DNA synthesis in these cells (Fig. 3c–e).

We were particularly interested in the result that average EdU intensity was similar between HaloTag-USP1-WT- and HaloTag-USP1-GG/AA-complemented cells, given our observations of fork elongation defects in the USP1-GG/AA cell lines. In considering the differences between the USP1 mutants, we speculated that relative Ub-PCNA levels in the HaloTag-USP1 complemented cells may play a role. The USP1 KO and USP1-C90S cells both have relatively high Ub-PCNA levels due to the absence of USP1 activity, and therefore increased potential for recruitment of lower fidelity TLS polymerases to the fork. By contrast, the USP1-GG/AA cells have relatively low Ub-PCNA levels, favoring the usage of the canonical replicative polymerases. We, therefore, surmised that although there is increased fork-stalling and asymmetric fork progression in these cells, as evidenced in Fig. 1c–e, the relatively low Ub-PCNA levels may allow for more efficient elongation from dormant or newly fired origins. To investigate this further, we measured replication fork speed of origins firing only during the second labeling period (red only tracts) in cells treated with or without PolK siRNA (Supplementary Fig. 2e). Interestingly, replication elongation from newly fired origins was similar between the HCT116 cell lines expressing USP1-GG/AA and USP1-WT, but significantly slower in the USP1 KO and USP1-C90S cell lines, presumably due to aberrant TLS polymerase recruitment. Strikingly, these fork speed defects could be rescued by siRNA-mediated depletion of PolK in the USP1 KO and USP1-C90S cells, further suggesting that unscheduled PolK recruitment may also contribute to slowing fork progression at these newly fired origins (Supplementary Fig. 2e). This increased TLS polymerase usage and resulting inefficient fork progression from dormant origins in the KO and C90S-complemented cells may partly explain the reduced EdU intensity in Fig. 3c–e.

We additionally labeled cells expressing the different HaloTag-USP1 constructs with the HaloTag ligand JF549 and analyzed the level of association of single USP1 molecules with EdU signal using Pair-correlation function analysis^36,38,39^ (see “Methods”, representative images and movies in Fig. 3g and Supplementary Movies 1, 2). Notably, cells that were mock-treated with the HaloTag JF549 ligand while not expressing HaloTag-USP1 protein did not produce significant background signal in our single-molecule imaging analysis (Supplementary Fig. 5a and Supplementary Movie 3). In quantifying the mean intensity of fluorescently-conjugated HaloTag signal for each of the different chromatin-bound USP1 mutants expressed per nucleus, we noted that the expression level of the HaloTag-USP1-C90S mutant was similar to that of HaloTag-USP1-WT, whereas the HaloTag-USP1-GG/AA density was slightly higher than the others (Supplementary Fig. 5a). Importantly, when we analyzed the average density of USP1 molecules around EdU by Pair-correlation function analysis, the highest correlation was observed in the HaloTag-USP1-GG/AA-complemented cells, indicating increased retention (trapping) of the GG/AA mutant proteins around sites of nascent DNA synthesis (Fig. 3h and Supplementary Fig. 5b). Our analysis of EdU density was not influenced by broad cell-to-cell variance in USP1 levels, as we found no apparent correlation between EdU density and USP1 signal intensity for each of the different HaloTag-USP1 mutants (Supplementary Fig. 5c). Taken together, these results suggest that aberrant USP1 engagement (increased density of USP1 molecules around EdU) at replication foci is a likely contributor to increased replication stress in the USP1-GG/AA mutant cells.

### Spartan mediates the removal of trapped USP1 mutants from DNA

We were intrigued by our findings that while the HaloTag-USP1-GG/AA mutant was aberrantly retained and localized at EdU foci, this “USP1-trapping” effect was not observed for the HaloTag-USP1-C90S mutant (Fig. 3h), even though both mutant proteins are incapable of autocleavage. Given data from recent publications showing that USP1-WDR48 complexes have DNA binding activity^19,20^, we first considered the possibility that perhaps the USP1 mutations could be affecting USP1’s recruitment to chromatin or the replication fork. To examine the chromatin binding status of each of the USP1 mutants, we prepared nuclear soluble and chromatin fractions of WT and USP1 mutant cell lines and quantified USP1 levels relative to WT-USP1 (Supplementary Fig. 6a). As anticipated, we observed a roughly three-fold increase in nuclear soluble levels of each of the USP1 mutants (C90S, GG/AA, and Q672V), reflecting their overall increased protein stability. Furthermore, we verified that each of the USP1 mutants were enriched in the chromatin fraction relative to WT (Supplementary Fig. 6a). Thus, the recruitment of each of the USP1 mutants to chromatin was not adversely impacted. Additionally, we compared the relative binding of USP1 to sites of nascent DNA synthesis in EdU-labeled, thymidine chase, and HU-treated samples by iPOND analysis, as performed previously^19^ (Supplementary Fig. 6b). From this study, we found that the USP1-GG/AA mutant was more enriched at nascent DNA in comparison to WT-USP1, but both were eventually removed from the DNA following an extended thymidine chase or treatment with HU (Supplementary Fig. 6b).

We next hypothesized that replication-coupled proteolytic activity may be selectively removing USP1-C90S molecules, while having minimal effect on the USP1-GG/AA mutant. The USP1-GG/AA and USP1-C90S cell lines are largely distinguishable by their DUB activity and effects on substrate deubiquitylation, particularly Ub-PCNA levels. Therefore, in considering cellular proteases that could process and thereby release “trapped” USP1 molecules from DNA, we deemed the DNA-dependent metalloprotease Spartan (SPRTN) to be a likely candidate, as it was previously characterized as a reader of PCNA ubiquitylation. As an orthologue of Wss1 in yeast^40^, SPRTN is a critical component of the DNA replication machinery in vertebrates that travels with elongating replication forks and degrades replication-blocking DNA-protein crosslinks (DPCs)^41–46^. DPCs can originate when proteins become inadvertently crosslinked to DNA after exposure to physical or chemical agents, such as UV light or aldehydes, or can result from faulty enzymatic reactions, such as with topoisomerases. Genomic instability and impaired fork progression arise in SPRTN-deficient human and mouse models due to the accumulation of DPCs that directly block efficient replication progression^41–43,45,47–49^. In addition to DPCs, SPRTN has also been shown to promote the clearance of tightly-bound proteins on DNA (non-covalent interactions) that act as DPC-like barriers. For example, a recent study demonstrated that SPRTN activity facilitates the removal of non-crosslinked, trapped PARP1-complexes from DNA following treatment of cells with PARP inhibitors^50^. Furthermore, SPRTN/Wss1 plays additional roles in the cleavage of non-crosslinked Chk1 from chromatin to promote ATR signaling^51^, and by degrading unassembled, non-covalently bound histones during replication stress^52^. We envisioned that SPRTN could be playing a similar role in promoting the clearance of trapped USP1 molecules.

**Fig. 4:**
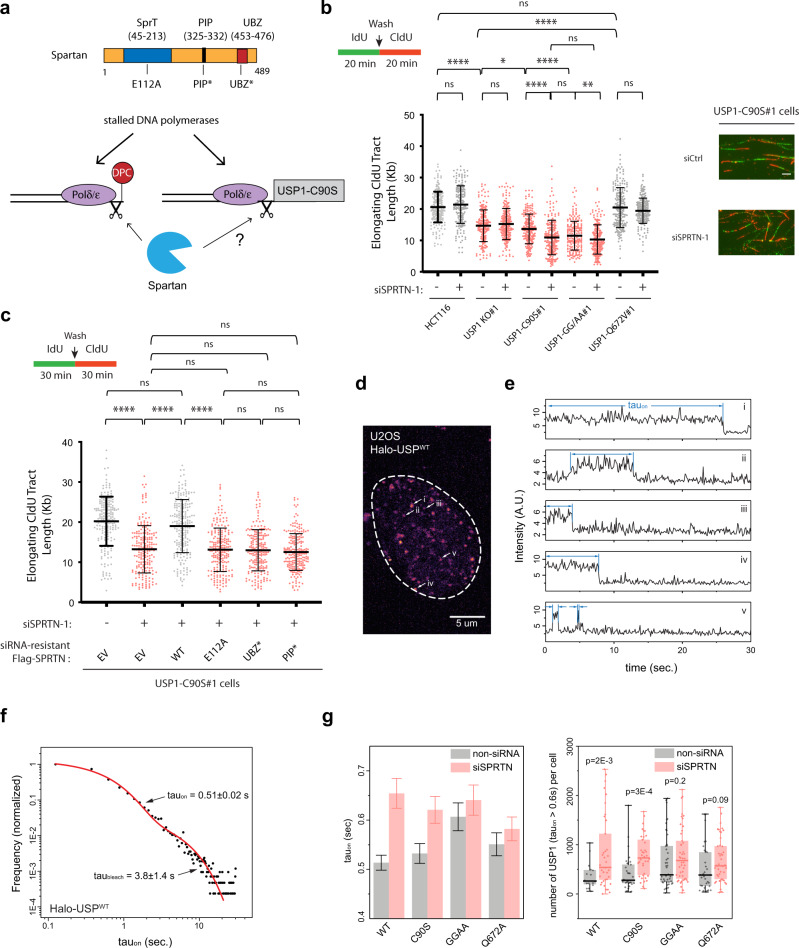
Spartan facilitates removal of USP1 from forks to enable replication elongation. **a** Schematic representation of Spartan-mediated removal of DPCs and tightly bound proteins during DNA replication. **b** (left) HCT116 cells were treated with either control or SPRTN siRNAs for 72 h, and subsequently pulse-labeled with IdU and then CldU for 20 min. CldU tract lengths were measured in each sample to assess relative speed of elongating forks. Data are plotted for three independent experiments (*n* = 200 elongating forks) with mean −/+ SD indicated and p values were calculated using the Mann–Whitney rank-sum t-test (ns = no significance, *p < 0.05, **p < 0.01, ****p < 0.0001, two-tailed). (right) Representative tracts of HCT116 USP1-C90S#1 cells treated with either siCtrl or siSPRTN-1. Scale bar = 5 μm. **c** HCT116 USP1-C90S cells were treated with either siCtrl or siSPRTN-1 siRNAs and complemented with the indicated siRNA-resistant Flag-SPRTN constructs. Following transfections, cells were pulse-labeled with IdU and then CldU for 30 min to assess fork speed of elongating forks. CldU tract lengths are plotted for three independent experiments (*n* = 200 elongating forks) with mean −/+ SD indicated and *p* values were calculated using the Mann–Whitney rank-sum t-test (ns = no significance, ****p < 0.0001, two tailed). **d** A representative maximum intensity projection image of Supplementary Movie 4 showing live-cell imaging of a U2OS cell expressing JF646-Halo-USP1-WT. Brighter spots indicate longer retention. **e** Representative intensity trajectories of the single USP1 molecules marked in (**d**). The dwell time (tau_on_) is labeled in blue. **f** Normalized distribution of tau_on_ for multiple USP1-WT molecules obtained from *N* = 16 nuclei. The distribution was fitted with two exponential decays, of which the faster decay corresponds to the average dwell duration of USP1 molecules, while the slower decay provides the photo-bleaching kinetics. **g** (Left) The average dwell duration of different USP1 molecules as indicated. Mean −/+ std is the fitting result and the std of the fitting of the distribution of tau_on_ built from *N* = 16, 33, 45, and 33 nuclei for WT, C90S, GG/AA, and Q672A, respectively. (Right) Number of molecules retained longer than 0.6 s in each cell with different USP1 expression as indicated (median: dark bar, box: 25–75% (1st and 3rd quartile), Whisker: minimum and maximum, *N* = 16, 33, 45, and 33 nuclei for WT, C90S, GG/AA, and Q672A, respectively. *P*-values were calculated with student’s t-test.

The recruitment of SPRTN to DNA damage sites and DPCs is modulated by binding to partner proteins, including p97 and PCNA. In addition to its putative metalloprotease SprT domain, SPRTN also contains a ubiquitin-binding zinc finger (UBZ) and PCNA-binding PIP box motif that preferentially binds ubiquitinated PCNA (Fig. 4a)^49,53^. Ectopic expression of only the full-length WT SPRTN, but not UBZ or PIP mutant forms of SPRTN, rescues detrimental effects of SPRTN silencing following formaldehyde treatment in human cells^54^. We postulated that the differences between PCNA ubiquitylation status between the different USP1 mutant cell lines could be affecting SPRTN-mediated proteolysis. Therefore, we decided to determine the effects of SPRTN knockdown on elongating fork speed in each of the USP1 mutant cells. In untreated USP1-WT, USP1 KO, and USP1-Q672V cells, SPRTN knockdown did not substantially affect elongating CldU tract lengths in comparison to control samples (Fig. 4b). This result is consistent with previous findings showing that SPRTN depletion only has a slight impact on fork speed in untreated HEK293T cells^45^. Interestingly, SPRTN knockdown caused a severe reduction in fork speed in USP-C90S cells, while having a more modest, but still significant effect on fork elongation in the USP-GG/AA cell line (Fig. 4b). Similar results were observed using an alternative siRNA oligo sequence against SPRTN (Supplementary Fig. 7a).

Additionally, we sought to determine whether the ability of SPRTN to remove trapped USP1 molecules is largely dependent on its protease activity and/or recruitment by Ub-PCNA. To examine these functional effects, we depleted endogenous SPRTN and complemented the USP1-C90S cells with siRNA-resistant forms of SPRTN-WT, a SPRTN-E112A mutant that disrupts protease activity^41^, as well as SPRTN mutants that disrupt either PCNA or Ub-binding (SPRTN-PIP* and SPRTN-UBZ*, respectively) (Fig. 4c and Supplementary Fig. 7b)^46^. Complementation of USP-C90S cells with SPRTN-WT rescued the fork speed defect observed upon siRNA-mediated SPRTN depletion, while expression of the catalytically-inactive SPRTN-E112A, SPRTN-UBZ*, and SPRTN-PIP* mutants did not, suggesting that both the protease activity and Ub-PCNA binding of SPRTN are critical for the processing and removal of trapped USP-C90S molecules on DNA (Fig. 4c and Supplementary Fig. 7b). Overexpression of the SPRTN-WT construct could not restore the fork speed defect in the USP1-GG/AA cells (Supplementary Fig. 7c); this is likely due to the already suppressed Ub-PCNA environment in these cells, especially if SPRTN requires Ub-PCNA binding to be fully functional for removal of USP1.

Given our findings of increased association of the HaloTag-USP1-GG/AA with nascent DNA (Fig. 3H), we wanted to determine how USP1 dynamics are affected by these mutations and upon knockdown of SPRTN. To monitor the nuclear dynamics and retention of individual USP1 molecules, we engineered U2OS cells stably-expressing HaloTag-USP1 fusions and labeled a small fraction of the Halo-USP1 with optimized concentration of JF646-Halo ligands for live-cell SMT as a function of USP1 mutations with or without SPRTN. HaloTag-USP1 fusions produced the expected USP1 autocleavage products and were engineered to be resistant to siRNA targeting of endogenous USP1 (Supplementary Fig. 7d). The U2OS cells also co-expressed a Cerulean-PCNA construct, which served as an S phase marker in live-cell analysis. As for the single-molecule localization analyses, mock treatment of U2OS cells with JF646 ligand without expression of HaloTag-USP1 fusions produced no detectable signal, indicating that background levels of HaloTag signal were not a confounding factor in our live-cell experiments (Supplementary Movie 3). Figure 4d shows a representative maximum intensity projection image of 1000 frames where the dynamics of WT-USP1 (JF646-HaloTag-USP1, Supplementary Movie 4) in single S-phase U2OS nucleus are shown. Here, the intensity of the spots corresponds to the average retention of individual USP1 molecules before they diffuse away or become photobleached. These movies displayed a clear difference in retention dynamics of USP1 mutants, revealing extended retention for USP1-GG/AA (Supplementary Movie 6) as compared to USP1-WT (Supplementary Movie 4) and USP1-C90S (Supplementary Movie 5). To quantify the retention kinetics of individual USP1 molecules, we analyzed their on-time duration, termed tau_on_, measuring their association with chromatin. Figure 4e shows representative intensity trajectories of the single USP1 molecules from Supplementary Movie 4 as marked in Fig. 4d, clearly showing their specific kinetics including persistent association (top trajectory) and more transient binding (bottom trajectory). We plotted the average distribution of tau_on_ for multiple USP1 molecules, which displayed two distinct kinetic processes whose curve fit yielded a lifetime at ~0.51 s and at ~3.8 s (Fig. 4f), of which the former represents the average retention duration of USP1 molecules tau_on_ while the latter is an independent factor attributed to their average photo-bleaching kinetics tau_bleach_^55^. In determining the number of HaloTag-USP1 localizations per frame over the course of the analyzed movies, we noted that the decay curves of HaloTag signal resulted only from the tau_on_ of the different USP1 mutants, and that the photo-bleaching kinetics was of similar magnitude between samples (Supplementary Fig. 7e). Analysis of the retention duration, tau_on_ for USP1-WT and USP1 with different mutations is shown in Fig. 4g (left graph), revealing that USP1-GG/AA mutant exhibited extended retention as compared to WT as well as other mutants. To provide an additional metric for the observed trends we also counted the number of USP1 molecules in each cell that resided longer than 0.6 s, which is the average dwell duration tau_on_ obtained for USP1-GG/AA. Consistent with the observed trend of tau_on_, this analysis showed an increase in the number of persistent USP1 molecules in cells expressing USP1-GG/AA mutant (Fig. 4g, right graph). Strikingly, depletion of SPRTN via siRNA resulted in altered USP1 kinetics as quantified via tau_on_ and measurements of persistent USP1 molecules (Fig. 4g), with USP1-WT and USP1-C90S mutant exhibiting prolonged duration of retention at similar levels to that of USP1-GG/AA mutant, indicating that SPRTN plays a critical role in the recycling of transiently interacting or trapped USP1 on DNA. However, the number of USP1 molecules residing longer than 0.6 s in the presence of SPRTN siRNA was much higher for the USP1-C90S and USP1-GG/AA mutants in comparison to USP1-WT (Fig. 4g, right). In contrast, the average tau_on_ for USP1-Q672A and USP1-GG/AA were not significantly affected by SPRTN knockdown; this is to be expected since the status of PCNA ubiquitylation is a critical component of SPRTN-mediated removal of DPCs (Fig. 4g). We note that, although only an optimized small fraction of the Halo-USP1 was labeled for SMT, we do not exclude the possibility that a few USP1 locus containing more than one fluorescently labeled USP1 molecule was taken into account and resulted in prolonged tau_on_. Hence the longer tau_on_ for USP1-GG/AA mutant may suggest either/both longer retention of individual USP1-GG/AA molecules or/and more USP1-GG/AA molecules residing on chromatin.

To further confirm the role of SPRTN in promoting the clearance of trapped USP1-C90S molecules, we additionally analyzed the chromatin recruitment of USP1 mutants in the presence or absence of siRNA-mediated SPRTN knockdown. As shown in Supplementary Fig. 7f, SPRTN knockdown resulted in a two-fold increase in the chromatin binding of USP1-C90S but did not as substantially impact the chromatin recruitment of the USP1-WT and USP1-GG/AA proteins. These results corroborate our DNA fiber analysis from Fig. 4b and live-cell imaging analysis from Fig. 4g suggesting that SPRTN selectively acts on the USP1-C90S mutant in particular to facilitate its clearance from DNA, while minimally affecting the chromatin binding of the other USP1 mutants.

### Replication stress and cell cytotoxicity caused by treatment with USP1-UAF1 inhibitor ML323 can be further sensitized by SPRTN loss

**Fig. 5:**
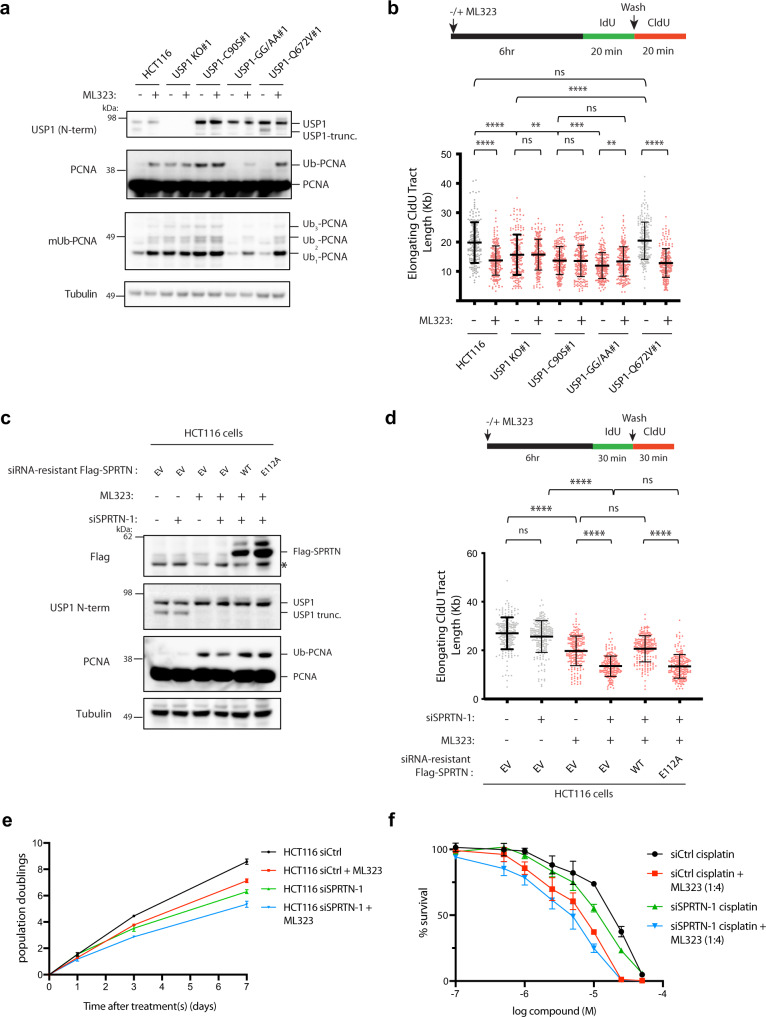
ML323 treatment prevents USP1 autocleavage and sensitizes cells to DNA replication and growth defects induced by SPRTN depletion. HCT116 cell lines were treated with or without 2.5 μM ML323 inhibitor for 6 h and subjected to either immunoblotting analysis in (**a**) or DNA fiber analysis of elongating fork speed (**b**). Data are graphed with mean and −/+ SD for 3 independent experiments (*n* = 200 elongating forks), and *p* values were calculated using the Mann–Whitney rank-sum t-test (ns = no significance, **p < 0.01, ****p < 0.0001, two-tailed). **c**, **d** HCT116 cells were treated with siRNAs for siCtrl or siSPRTN-1 and transfected with the indicated siRNA-resistant Flag-SPRTN constructs. Cells were then treated with or without 2.5 μM ML323 and subjected to immunoblotting analysis in (**c**) or DNA fiber analysis for elongating fork speed (**d**). CldU tract lengths are plotted for three independent experiments (*n* = 200 elongating forks) with mean −/+ SD indicated and *p* values were calculated using the Mann–Whitney rank-sum t-test (ns = no significance, ****p < 0.0001, two-tailed). **e** HCT116 cells were transfected with either siCtrl or siSPRTN-1 siRNAs and treated with or without 2.5 μM ML323 inhibitor. Cell population doublings were calculated at 3 and 7 days for three independent experiments, with mean −/+ SD indicated. **f** Cytotoxicity assay of control or SPRTN-depleted HCT116 cells, treated with either cisplatin alone or a combination of cisplatin and ML323 at a ratio of 1:4. The data represent the mean ± SD of three independent experiments.

A chemical inhibitor of USP1, ML323, has been developed that showed promise in inducing cytotoxicity in cisplatin-treated non-small lung cancers and osteosarcoma cells^56^. We were therefore interested in determining whether treatment of HCT116 cells with this inhibitor mimics the fork speed defect observed in the USP1-C90S cells by DNA fiber analysis. Treatment of cells with the ML323 inhibitor for 6 h was sufficient to block the catalytic activity of USP1 in cell lines with active USP1/UAF1 complexes, as evidenced by induction of PCNA monoubiquitylation and prevention of USP1 autocleavage (Fig. 5a). In agreement with its on-target specificity, while ML323 treatment had no significant effect on fork speed in USP1 KO and C90S cells, it significantly reduced elongating tract lengths in cells expressing WT and Q672V-USP1 (Fig. 5b). Notably, treatment of the USP1-GG/AA cells with ML323 failed to fully recover PCNA ubiquitylation back to WT levels and resulted in a slight rescue in the fork speed defect to the level of the USP1-C90S cells (Fig. 5a, b). A likely explanation for this result could be because the GG/AA mutant possess a stronger retention on DNA (slower recycling kinetics) (Fig. 4g) than either WT or the Q672V mutant, this may cause a steric hindrance on the recruitment of Rad6-Rad18 (ubiquitin E3 ligase) to efficiently ubiquitylate PCNA within the 6 h ML323 treatment time-frame. Since ML323-treated USP1-GG/AA cells can still partially upregulate PCNA ubiquitylation, this may account for some minimal SPRTN recruitment and clearance of trapped GG/AA molecules at the fork. Nevertheless, ML323 treatment of HCT116 parental cells phenocopies USP1-C90S cells in its effects on reducing replication fork speed.

As the combination of SPRTN knockdown in the C90S cells caused a dramatic reduction in fork speed (Fig. 4b), we were curious to determine whether ML323 treatment combined with SPRTN knockdown would cause similar effects as the C90S cells. While SPRTN knockdown on its own had little effect on replication fork speed, treatment with ML323 for 6 h led to a reduction in fork speed in HCT116 cells (Fig. 5c, d). Intriguingly, depletion of SPRTN in ML323-treated cells led to an even greater reduction in fork speed, which could be rescued by transfection with siRNA-resistant WT-SPRTN, but not the catalytically inactive E112A mutant (Fig. 5c, d). Thus, much like in the USP1-C90S cells, loss of SPRTN activity in ML323-treated cells caused a dramatic reduction in replication fork elongation, which is likely due to the buildup of ML323-induced retention or “trapping” of full-length USP1 molecules on DNA.

Finally, to examine cellular consequences of SPRTN depletion and ML323 treatment, we compared effects of these treatments on cell proliferation. Over the course of 7 days, treatments with either ML323 or SPRTN siRNA alone had only modest effects on cell growth (Fig. 5e). However, replication challenges incurred from combined ML323 and SPRTN treatment resulted in greater inhibition of cell proliferation rates (Fig. 5e). Considering this result, we surmised that these replication and growth defects incurred upon combined SPRTN depletion and ML323 treatment would further exacerbate cytotoxicity induced by chemotherapeutics and crosslinking agents such as cisplatin. A strong synergistic effect between cisplatin and ML323 had been previously demonstrated to inhibit the proliferation of H596 and U2OS cells^56^. We, therefore, compared HCT116 cells treated with either siSPRTN, ML323, or both for their sensitivities to varying doses of cisplatin. We found that ML323 treatment sensitized HCT116 cells to cisplatin-induced cytotoxicity, and this effect was further exacerbated by siRNA-mediated knockdown of SPRTN (Fig. 5f). Thus, aberrant retention of USP1 molecule on DNA caused by combined SPRTN and USP1 catalytic inhibition may be effective in further enhancing cytotoxicity of cisplatin and other chemotherapeutics in a variety of cancer cells.

## Discussion

**Fig. 6:**
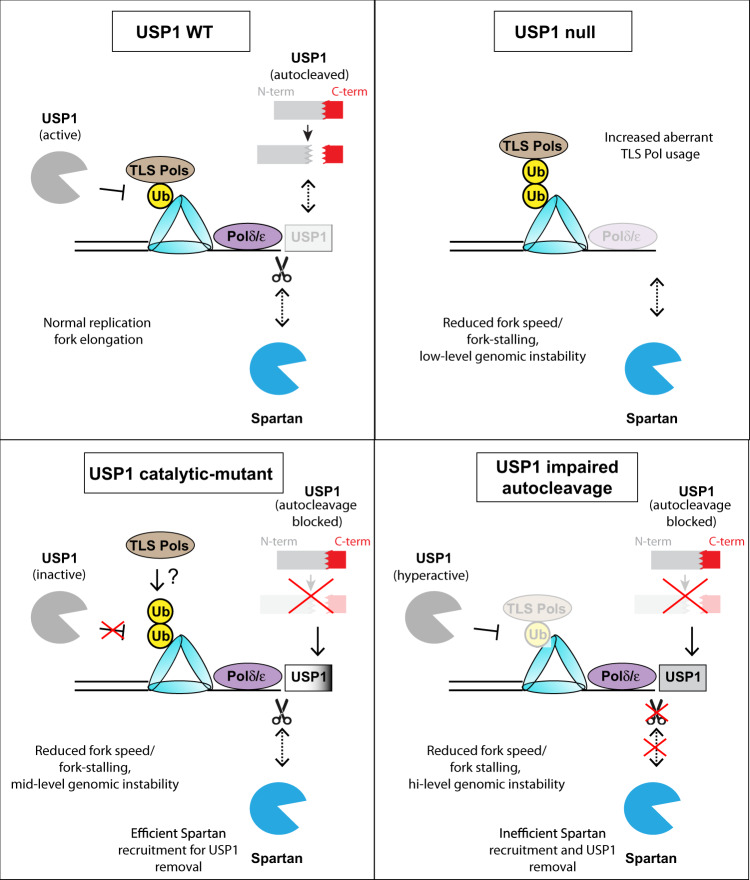
Model for the role of USP1 activity and/or autocleavage during normal DNA replication progression. During unperturbed replication, low levels of PCNA ubiquitylation allow for efficient TLS to overcome various replicative lesions, and autocleavage allows USP1 to be dynamically recruited to the fork. By contrast, the complete absence of USP1 activity leads to increased aberrant TLS Pol usage due to hyperubiquitylation of PCNA, resulting in reduced replication fork speed and low-level genomic instability. Impaired USP1 autocleavage caused by mutations disrupting the catalytic activity (C90S) or autocleavage site (GG/AA) additionally causes increased fork stalling and replication stress due to increased retention (i.e., “trapping”) of USP1 on DNA. The DNA-metalloprotease SPRTN is efficiently recruited to remove the trapped USP1 catalytic-mutant to mitigate these effects, owing to high Ub-PCNA levels in cells expressing this form of USP1. USP1 hyperactivity and deubiquitylation of PCNA in cells harboring the autocleavage-deficient GG/AA mutant, on the other hand, does not allow for effective SPRTN-mediated removal, resulting in high-level USP1 trapping. Thus, we propose that proper regulation of USP1 activity and autocleavage are both important for ensuring normal USP1 dynamics at the fork and genome stability.

Overall, our study provides a working model whereby USP1 autocleavage potentiates its dynamic association with the replication fork to enable proper replication fork elongation and genome stability (Fig. 6). In unperturbed conditions, USP1 likely travels with the elongating fork to periodically deubiquitylate PCNA (and potentially other substrates) to ensure proper replication timing. However, upon replication stress, USP1 becomes autocleaved to enable its displacement from stalled forks, allowing for a transient increase in PCNA ubiquitylation to allow for efficient TLS and other replication stress responses. Fitting with the model we proposed previously^18^, USP1 KO cells are characterized by PCNA hyper-ubiquitylation (due to the complete absence of USP1), which in turn, promotes aberrant usage of error-prone TLS polymerases. Increased recruitment of TLS polymerases results in heightened fork-stalling and slowed/delayed replication fork progression, leading to genomic instability (Fig. 6). Interestingly, in this study, we found that while USP1-C90S mutant cells phenocopies the USP1 KO cells, as characterized by PCNA hyper-ubiquitylation, depletion of TLS polymerases in the USP1-C90S cells fail to alleviate this fork-stalling defect. The difference is likely due to its inability to be autocleaved and to be efficiently removed from DNA during DNA replication. It is unclear if the initial engagement of the C90S mutant with PCNA may block TLS polymerase recruitment, explaining the inability to rescue the fork speed defect in these cells through depletion of TLS polymerases. However, the full-length trapped USP-C90S molecules are able to be efficiently removed from DNA by SPRTN-mediated proteolysis. This is facilitated by the recruitment of SPRTN to stalled forks by the Ub-PCNA signal (Fig. 6). By contrast, due to suppressed Ub-PCNA levels in USP1-GG/AA cells, SPRTN is unable to be recruited and properly displace the USP-GG/AA mutant from replication forks, causing USP1 complexes to be aberrantly retained and block proper fork progression (Fig. 6). In summary, by using different USP1 knock-in mutants in HCT116 cells and single-molecule imaging tools, we have revealed a new role for USP1 autocleavage in modulating USP1-replication fork engagement to ensure proper replication progression and maintenance of genome integrity in cells (Fig. 6).

The regulation of USP1 by autocleavage draws some interesting parallels to other proteases that self-inactivate by cleaving themselves, including SPRTN itself. While binding of SPRTN to ssDNA triggers proteolysis of substrates, dsDNA binding by SPRTN specifically induces its autocatalytic activity, which serves as a safeguarding mechanism to enable release of the enzyme from chromatin and limit unwanted proteolysis^41,44^. Experiments using fluorescence recovery after photobleaching (FRAP) revealed that while SPRTN-WT and a catalytically-inactive SPRTN mutant (SPRTN-EQ) are recruited to DNA damage sites equivalently, there is a much slower recovery after bleaching for the SPRTN-EQ mutant, indicating that it is more stably associated with the damage site once recruited^44^. Our experiments using live-cell imaging analysis of HaloTag-USP1 fusions similarly indicate that the USP1-GG/AA mutant, which is unable to be autocleaved or efficiently processed by SPRTN, has a slow diffusion rate in comparison to the other HaloTag-fusions tested in untreated conditions (Fig. 4g). Furthermore, siRNA-mediated depletion of SPRTN resulted in increased dwell time and number of retained USP1 molecules for all samples, particularly for the autocleavage-deficient USP1-C90S mutant (Fig. 4g). Recently, it was shown that the autocatalytic inactivation of SPRTN is controlled by a ubiquitin switch in a E3 ligase-independent manner, whereby monoubiquitylation induces cleavage of SPRTN in *trans* while also priming the enzyme for proteasomal degradation^57^. Whether a similar ubiquitin switch regulates USP1, as well as elucidation of the upstream triggering mechanism for USP1 self-cleavage, will be interesting topics for future investigations.

Another important unanswered question is how the defect in USP1 autocleavage promotes increased retention of USP1 on nascent DNA. Based on sequence alignments with USP12, which does not bind DNA, a recent study by D’Andrea and colleagues identified critical DNA-binding regions within USP1 that were deleted to generate a USP1 mutant that fails to bind DNA (USP1-Trunc)^19^. Intriguingly, this USP1-Trunc mutant lacks the autocleavage site in full-length USP1, indicating that this site and surrounding residues may be important for USP1 binding to DNA^19^. Additionally, a newly identified PIP motif within the L1 insert of USP1 was shown to be important in mediating the interaction of USP1 with PCNA, and a double mutant lacking both DNA and PIP interactions was severely compromised in its ability to deubiquitinate DNA-loaded PCNA^58^. Thus, it is tempting to speculate that this PIP motif and L1 region cooperate with the autocleavage site region for DNA-binding interactions of USP1. A recent publication features the crystal structure of human USP1-UAF1 complex with and without ubiquitin, and one that is bound to monoubiquitinated FANCI-FANCD2^59^. The GG (670–671) autocleavage site lies within the L2 insert region, which was omitted from the structure due to difficulty in obtaining crystals that diffract at <4 Angstroms. However, it is interesting to note that insert 1 and 2 regions are positioned on the opposite face of USP1 to the ubiquitin-binding site in this structure, indicating that these regions are less important for catalyzing deubiquitylation^59^. Thus, the autocleavage site may be less important for deubiquitylation of substrates, and more involved in controlling USP1-DNA binding dynamics.

Finally, our study sheds additional light on the mechanism by which the USP1 inhibitor ML323 derives its cytotoxicity when used in combination with chemotherapeutics such as cisplatin. Previously, the cytotoxic effects of ML323 treatment were linked primarily to increasing the monoubiquitylation levels of PCNA and FANCD2, targets of the TLS and FA pathways that mediate tolerance to cisplatin-induced inter- and intra-strand crosslinks^56^. Here, we further show that ML323 treatment also prevents USP1 autocleavage, causing increased numbers of USP1 molecules to become inadvertently trapped on DNA. Thus, catalytic inhibitors of USP1 (such as ML323) may be a means to induce additional replication stress in cancer cells via this ‘USP1-trapping’ mechanism. Additionally, considering our result that combined SPRTN depletion and ML323 treatment augments the cytotoxicity induced by cisplatin (Fig. 5f), this raises the possibility that the expression level of SPRTN (and possibly other DNA-dependent proteases) may be a useful biomarker to determine the potential effectiveness of using USP1 inhibitors in cancer treatment. It is not yet clear where exactly ML323 binds to USP1 and by what mechanism the inhibitor prevents DUB activation and induces this ‘USP1 trapping’ effect. Recently, broad classes of DUB inhibitor types have been conceptualized based on their mode of inhibition, specifying whether a particular drug targets the DUB active site (Type I), stabilization of an inactive DUB conformation (Type II), allosteric Ub binding sites of a DUB (Type III), allosteric sites outside of Ub-binding sites (Type IV), or multiple regions of a DUB simultaneously (Type V)^60^. Additional structural information modeling interaction sites for ML323 (as well other USP1 inhibitors) will help define which class of DUB inhibitors this drug falls into and will be instrumental moving forward to improve strategies for selectively targeting the UAF-USP1 complex and potentially other DUBs therapeutically.

## Methods

### Key resources and reagents

Key resources and reagents used in this study are described in Supplementary Table 1.

### Tissue culture

HCT116 cells were cultured at 37 °C in McCoy’s 5A media (Gibco) supplemented with 10% FBS (Atlantic Biologicals), 1% penicillin/streptomycin (Gibco), and 1% glutamine (Gibco). U2OS cells expressing HaloTag-USP1 fusion proteins and HEK293T cells were cultured at 37 °C in DMEM (Gibco) supplemented with 10% FBS (Atlantic Biologicals), 1% penicillin/streptomycin (Gibco), and 1% glutamine (Gibco). All cell lines were tested for mycoplasma using the Roche MycoTOOL Detection.

### Transfections of plasmids, siRNAs, and CRISPR sgRNAs

To generate USP1 biallelic knock-in mutations in HCT116 cells, two of the following gRNAs for each mutation site were individually cloned into a bicistronic vector for co-expression with the Cas9 protein. Based on the sequence of the selected gRNAs (see gRNA sequences in Supplementary Table 1), single-stranded oligodeoxyribonucleotide (ssODN) donors were co-transfected to generate the desired point mutations (see donor DNA sequences in Supplementary Table 1). Following vector delivery into target cells, individual clones were selected and evaluated by Illumina sequencing for verification (performed by Applied Stem Cell technologies).

Transient plasmid transfections and siRNA transfections were performed using Lipofectamine 3000 (Invitrogen) and Lipofectamine RNAimax (Invitrogen) reagents, respectively, according to the manufacturer’s instructions. Downstream analyses were performed 24 h following plasmid transfections and after 72 h of siRNA knockdown. pBabe_puro_HaloTag and pBabe_puro_NLS_mCerulean-PCNA vectors were gifts from the E. Rothenberg and M.Pagano labs, respectively. USP1-WT and mutant constructs were generated by PCR and subcloned into pHTn-HaloTag (Promega) and pBabe-puro-HaloTag vectors. Retroviral packaging of HaloTag-USP1 fusions and NLS_mCerulean-PCNA was performed using standard protocols in HEK293T cells with pVSVG and pGP packaging vectors followed by infection of U2OS cells. Stable cell lines expressing HaloTag-USP1 fusions and NLS_mCerulean-PCNA were selected by labeling with DirectTMR HaloTag ligand (Promega) and FACS sorting (assisted by NYU Cytometry & Cell Sorting Laboratory). pcDNA3 Flag-Spartan WT and E112A vectors were purchased from Addgene (#110214 and #110215, respectively)^42^. siRNA target sequences (Qiagen) used in this study are as follows:

All-Star Negative Control siRNA; PolK siRNA (siPolK-5): #5, 5′-TGGAATTAGAACAAAGCCGAA-3′; USP1 siRNA (siUSP1-1): #1 5′-TCGGCAATACTTGCTATCTTA-3′; Spartan siRNA (siSPRTN1 and −7): #1 5′-AGCCAATATAACGGTATACCA-3′, #7, 5′- ACAGTTGGCAACATCCCTAAA-3′.

USP1 and Spartan constructs resistant to siRNA-mediated downregulation were engineered to contain synonymous-codon mutations, indicated by upper-case below: USP1 siRes: #1: 5′-tTggAaaCacGtgTtaCTtGa-3′, #2 (used with C90S mutant): 5′- tTggAaaCacGagttaCTtGa-3′

Spartan siRes: 5′- ggagcAaaCatCacCgtG-3′. Primers used for site-directed mutagenesis are as follows: USP1_siResist_1_F: 5′-GTGGGACTGAATAATCTTGGAAACACGTGTTACTTGAATAGTATACTTCAGG-3′; USP1_siResist_2_F (for C90S mutant): 5’GTGGGACTGAATAATCTTGGAAACACGAGTTACTTGAATAGTATACTTCAGG-3′;

Spartan_siResist_F: 5′- CATCAACAGCCTGACTGGAGCAAACATCACCGTGTACCATACTTTTCACGATG-3′;

Spartan_UBZ_F (C456G, C459G): 5′-AGCAAAATGGTTAATGGCCCAGTTGGTCAGAATGAAGTTCTG-3′;

Spartan_PIPmut_F (Y331A, F332A): 5′-AATGTTCTAAGCAACGCCGCTCCTAGAGTATCATTTG-3′.

### DNA fiber analysis

DNA fiber analysis was performed as described previously^38,39^. Briefly, cells were labeled sequentially with 50 μM IdU and CldU for the times indicated in each experiment. Following trypsinization, cells were washed and resuspended in cold PBS at a density of 1 × 10^6^ cells/ml. Cells were then spotted onto a glass slide and lysed in lysis buffer (0.5% SDS, 200 mM Tris-HCl (pH 7.4), 50 mM EDTA) for 6 min. Slides were tilted at a 15 °C angle to allow DNA fibers to spread by gravity flow and fixed for 3 min in chilled 3:1 methanol: acetic acid solution. The DNA was denatured using 2.5 N HCl, washed in PBS, and incubated in blocking buffer (5% BSA in PBS with 0.1% Triton X-100) prior to staining with primary antibodies (mouse anti-IdU [B44] (BD Biosciences 347580, 1:150 dilution) and rat anti-CldU [BU1/75 (ICR1)] (Abcam ab6326, 1:200 dilution)) and fluorescent secondary antibodies (Goat anti-mouse IgG (H+L) Alexa Fluor 488 (Thermo Fisher A11001, 1:350 dilution) and Goat anti-Rat IgG (H+L) Alexa Fluor 594 (Thermo Fisher A11007, 1:350 dilution)). Stained slides were imaged using a Keyence BZ-X710 microscope. A minimum of 200 fibers were analyzed for each independent experiment assessing fork speed (CldU track length), and analysis shows the pool of three biological replicates per condition. Track lengths were calculated by converting μM measured in ImageJ to kb using the following conversion: 1 μm = 2.59 kb^61^.

### Lysis conditions and immunoblotting

For direct analysis by immunoblotting, cells were routinely lysed in denaturing SDS buffer (100 mM Tris (pH 6.8), 2% SDS, and 20 mM β-mercaptoethanol), and cell extracts were separated on NuPAGE 4–12% Bis-Tris or 3–8% Tris-Acetate gels (Invitrogen). Proteins were transferred onto 0.45 μm PVDF membrane (Millipore). Membranes were blocked in 5% milk in TBS-T for 1 h prior to incubation with primary antibody overnight. The following day, membranes were incubated with HRP-conjugated secondary antibodies (Peroxidase AffiniPure Goat Anti-Mouse IgG (H+L) (Jackson Labs 115-035-003, 1:10,000 dilution) and Peroxidase AffiniPure Goat Anti-Rabbit IgG (H+L) (Jackson Labs 111-035-003, 1:10,000 dilution)) in 5% milk in TBS-T and developed using ECL Prime reagent (GE Healthcare). Antibodies were purchased from the following sources for immunoblotting: USP1 (Bethyl A301-698A, 1:3000 dilution) MCM2 (Bethyl A300-191A, 1:10,000 dilution) PCNA [PC10] (Abcam ab29, 1:3000 dilution), Chk1 (Abcam ab2845, 1:1000 dilution), Histone H3 (Abcam ab1791, 1:10,000 dilution) Ubiquityl PCNA (Lys 164) [D5C7P] (Cell Signaling Technologies 13439S, 1:1000 dilution), Chk1 (Phospho Ser345) [133D3] (Cell Signaling Technologies 2348S, 1:5000 dilution), α-Tubulin [DM1A] (CalBiochem CP06, 1:10,000 dilution), Polκ (Santa Cruz Biotechnology sc-166667, 1:1000 dilution), MCM7 (Santa Cruz Biotechnology sc-9966, 1:5000 dilution), and Flag [M2] (Sigma-Aldrich F1804, 1:5000 dilution).

### DUB activity assay

USP1 activity was profiled in cell extracts using the activity-based probe Ub vinyl sulfone (Ub-VS) (Boston Biochem) as previously described^62^. HCT116 cells from a 6 cm dish per condition were lysed in DUB lysis buffer (50 mM Tris (pH 8.0); 50 mM NaCl; 5 mM DTT; 0.2% Triton X-100; 50 μM PMSF), and extracts were clarified by centrifugation at 14,000 × *g* for 15 min at 4 °C. Lysates were incubated with or without 0.5 μM HA-tagged Ub-VS (Boston Biochem) for 1 h at 37 °C with gentle agitation. Reactions were quenched with 1X SDS loading buffer (5% SDS, 25% glycerol, 150 mM Tris (pH 6.8), 200 mM DTT) prior to loading on NuPAGE gels and immunoblotting.

### Flow cytometry analysis

Cells were pulse-labeled with 10 uM EdU prior to trypsinization and fixation in 4% paraformaldehyde. EdU incorporation was detected using the Alexa Fluor 594 Click-iT EdU Flow Cytometry assay kit (Thermo Fisher Scientific) and DNA was counterstained with 1 μg/ml DAPI (Molecular Probes) prior to analysis. Flow cytometry data acquisition was performed on an LSRII (BD Biosciences) and data was analyzed using FlowJo software (Treestar, Ashland, OR).

### Chromatin fractionation

Cells were harvested from one 6 cm dish per condition. Chromatin fractionations were performed using a Subcellular Protein Fractionation Kit (Thermo Scientific) according to the manufacturer’s instructions. Densitometry analyses of immunoblots were carried out using Image J software (NIH).

### iPOND

iPOND was performed as described previously^20,38,63^. Briefly, six 15 cm dishes of HCT116 cells were pulse-labeled for 10 min with 10 μM EdU and then either collected directly or washed and treated with 10 μM thymidine for 1 h or 2 mM HU for 3 h. Cells were then cross-linked in 1% formaldehyde in PBS for 20 min at RT, and cross-linking reactions were quenched using 1 mL of 1.25 M glycine. Following permeabilization in 0.25% Triton X-100 for 30 min at RT, cells were washed and resuspended in click reaction buffer (2 mM CuSO_4_, 10 mM sodium ascorbate, and 10 μM biotin azide (Invitrogen) in PBS) for 2 h. Cell pellets were washed, lysed in SDS lysis buffer (50 mM Tris (pH 8.0), 1% SDS with protease inhibitor tablet (Roche)), and sonicated for 20 s at 40% amplitude for a total of 5 pulses. Insoluble material was removed by centrifugation, and lysates were transferred to a new tube and diluted 1:1 with PBS. Lysates were incubated for 3 h at 4 °C with 25 μl of streptavidin agarose beads (EMD Millipore). Beads were washed twice with cold lysis buffer, once with 1 M NaCl, and twice more with cold lysis buffer, prior to elution in 2X SDS loading buffer (5% SDS, 25% glycerol, 150 mM Tris (pH 6.8), 200 mM DTT) for 25 min at 95 °C. Subsequent SDS-PAGE and immunoblotting were performed as previously described.

### Ok-seq library preparation

Thirty 15 cm dishes of exponentially growing HCT116 parental and mutant cells of 60–70% confluency were labeled with EdU for 2 min at 25 μM final concentration. Okazaki fragments were purified and the libraries were generated as described previously^32^. Frozen cell pellets were lysed by mixing gently in DNA lysis buffer (10 mM Tris, pH = 8, 25 mM EDTA and 100 mM NaCl) with 0.5% (vol/vol) SDS and 0.1 mg ml^−1^ proteinase K overnight at 50 °C. DNA was extracted using an equal volume of phenol-chloroform and precipitated with ammonium acetate and ethanol. Following successive washes with 70% ethanol, DNA was isolated and resuspended in Tris-EDTA (TE) at 4 °C overnight. DNA was denatured at 95 °C for 10 min and chilled on ice for 10 min before undergoing size-fractionation on a neutral sucrose gradient with a Beckman Coulter Optima XE-100 (check the model) with SW32TI rotor at 122,000 × *g* at 20 °C for 17 h, with maximum acceleration and minimum deceleration. Size separation of genomic DNA was assessed using an alkaline 1.5% agarose gel and only fractions containing DNA fragments ≤200 bp were collected and concentrated. Subsequently, EdU-labeled DNA was biotinylated in a Click-it reaction (10 mM Tris-HCl pH 8, 2 mM CuSO_4_, 10 mM sodium ascorbate, and 2 mM Biotin TEG Azide (Berry Associate). Purified DNA was then subjected to RNA hydrolysis with 250 mM NaOH at 37 °C for 30 min and later phosphorylated with T4 polynucleuotide kinase (NEB). Ligation of adaptors was then performed in a two-step procedure, before and after pulldown of biotinylated DNA using MyOne T1 Streptavidin dynabeads (Thermo Fisher Scientific) in 10 mM Tris pH 7.5, 1 mM EDTA, 2 M NaCl, and 0.1% (vol/vol) Tween 20 Buffer. Okazaki fragment libraries were generated by PCR amplification and primer dimers were removed using SPRI beads (Beckman) before analysis by Tapestation (Agilent). Libraries were sequenced (paired-end 50 bp) using the NovaSeq 6000 Illumina sequencing platform and SP100 flowcells.

### Ok-seq data analysis and statistics

Sequenced reads were aligned to the human genome (hg19 index) using bowtie2 and the read counts for Watson and Crick strands were binned into 1 kb bins for analysis. To assess origin firing localization and efficiency around genomic loci, TSS and TTS locations were first obtained from the UCSC genome browser (http://genome.ucsc.edu/cgi-bin/hgTables). Python scripts were used to produce plots of strand bias around gene TSS and TTS. Genes were separated by quartile based on RNA-seq read density (FPKM) for examining the relation between transcription and replication (FPKM values were obtained from ref. ^64^). Normalization by gene length was also used to visualize strand bias over the entire gene region. Statistics and effect sizes to test for significant difference between strand bias curves were calculated by taking the difference between ranges downstream and upstream of the site being analyzed. These ranges were: 1–10 kb downstream and 50–30 kb upstream of annotated TSS and ±1–10 kb around annotated TTSs. *P* values were calculated using the non-parametric Kruskal–Wallis H-test for indicated gene lists, testing the null hypothesis that the population medians of all groups are equal.

### Immunofluorescence

For 53BP1/Cyclin A staining, cells on coverslips were fixed for 15 min in 4% paraformaldehyde followed by 5 min in ice-cold methanol. Fixed cells were then permeabilized in 0.5% Triton X-100, blocked for 1 h in 2% BSA, 0.2% Triton X-100 at RT, and incubated for 2 h at RT with primary antibodies (rabbit anti-53BP1 (Abcam ab175933, 1:200 dilution) and mouse anti-Cyclin A2 (Calbiochem CC17, 1:100 dilution)). Coverslips were washed in PBS, incubated for 45 min in secondary antibodies (Goat anti-mouse IgG (H+L) Alexa Fluor 546 (Thermo Fisher A11003, 1:350 dilution) and Goat anti-rabbit IgG (H+L) Alexa Fluor 488 (Thermo Fisher A11008, 1:350 dilution)) washed again with PBS, and mounted onto slides using Vectashield with DAPI. Imaging was performed using Keyence BZ-X710 microscope. A minimum of 300 Cyclin A-negative cells were analyzed for 53BP1 foci from three independent experiments.

### Multi-color single-molecule localization microscopy (SMLM)

For cell preparation of EdU imaging experiments, HCT116 USP1 KO cells transfected with the indicated HaloTag-USP1 fusions were labeled for 15 min with 20 nM JF549 (Promega) followed by 10 μM EdU for 10 min. Cells were pre-fixed on coverslips with 4% paraformaldehyde for 60 s, and extracted using CSK buffer (10 mM Hepes (pH 7.4), 300 mM sucrose, 100 mM NaCl, 3 mM MgCl_2_, and 0.5% Triton X-100) for 10 min at RT. Following extraction, cells were washed 3 times with PBS and fixed in 4% paraformaldehyde for 15 min at RT. EdU was then detected with AlexaFluor647 (AF647) conjugated picolyl azide via the commercial click-iT reaction kit (ThermoFisher, C10640). Coverslips with cells were then blocked at 4 C overnight in PBS buffer with 2% BSA, 2% glycine, 0.2% gelatin, and 50 mM NH_4_Cl. Coverslips were mounted onto microscope glass with home-made buffer channels, which are filled with freshly mixed imaging buffer (1 mg/mL glucose oxidase, 0.02 mg/mL catalase, 10% glucose, and 100 mM cysteamine) during super-resolution (SR) imaging.

The SR imaging was performed on a home-built imaging platform equipped with a custom-conFig.d ASI microscope and other optical elements. In brief, a 639 nm laser (ultralasers, MRL-FN-639-800) and a 561 nm laser (Coherent, Sapphire 561 LPX-500) was aligned, expanded, collimated, and directed into an TIRF objective (Olympus, UApo N, 100x NA1.49) by a penta-edged dichroic beam splitter (Semrock, FF408/504/581/667/762-Di01). The lasers were adjusted to a Highly Inclined and Laminated Optical sheet (HILO) illumination mode with the illumination intensity at ~1.5 and 1.0 kW/cm2 for the 639 and 561 nm, respectively, at the exit of the objective. A 405 nm laser (ultralasers, MDL-III-405-100) was used to drive AF647 to its ground state. AF647 and JF549 were sequentially illuminated by the 639 and 561 nm laser, respectively. Each of their fluorescence was expanded (1.67×) and filtered by a single-band pass filter (Semrock, FF01-676/37 and FF01-607/36 for AF647 and JF549, respectively). The filters were switched via a filter wheel (ASI, FW-1000) for sequential illumination. Photons were then collected on a sCMOS camera (Teledyne Photometrics, Prime 95B) at 33 Hz (30 ms per frame) for 2000 frames.

Single-molecule localization was performed following a regular DAOSTORM routine^65^. Each frame of the raw image stack was first filtered with a small (σ ~ = 143 nm) and a bigger (σ ~= 286 nm) Gaussian kernel. The local maximum was then identified by subtracting the latter from the former filtered images. Note that the filtering was weighted by the inverse of the pre-calibrated pixel-dependent variance^66^. A 9 × 9 square (1 pixel ~= 65 nm) around each local maximum was cropped and submitted for MFA sub-pixel localization, which is achieved by fitting the data to one or more 2D-Gaussian point spread functions (PSF) through Maximum Likelihood Estimation (MLE). In Brief, the likelihood function is constructed by convolving the Poisson distribution parameterized by the photons emitted from a fluorophore nearby and the Gaussian distribution of the pre-calibrated readout noise of the camera. The number of fluorophores was increased in a Push-and-Pull manner to maximize the likelihood function^67^. The fitting accuracy of each emitter given by the Cramér-Rao Lower Bound (CRLB) was analyzed by fitting its distribution with a skew-Gaussian distribution, of which the center is used as the average localization precision. For pair-correlation analyses, localizations appeared within 2.5 times of the average localization precision in consecutive frames was averaged and considered as one localization from one blinking event. The coordinates list was then directly submitted for pair-correlation analyses. The representative images in Fig. 4d were rendered from the coordinate list to the 10 nm pixel canvas and blurred with a Gaussian kernel (σ = 10 nm) for display purpose.

### Alignment of the two-color channels

Alignment of the two color-channels was performed as described previously^68^. Pan-spectrum fluorescence beads (TetraSpec, T-7279, ~100 nm Diameter) were imaged in both channels, and their localization vectors ${{{{{{\bf{x}}}}}}}^{{{{{{\rm{AF}}}}}}647}={\left[{x}_{1}^{{{{{{\rm{AF}}}}}}647},{x}_{2}^{{{{{{\rm{AF}}}}}}647},\cdots ,{x}_{i}^{{{{{{\rm{AF}}}}}}647},\cdots ,{x}_{N}^{{{{{{\rm{AF}}}}}}647}\right]}^{{{{{{\rm{T}}}}}}}$, ${{{{{{\bf{y}}}}}}}^{{{{{{\rm{AF}}}}}}647}={\left[{y}_{1}^{{{{{{\rm{AF}}}}}}647},{y}_{2}^{{{{{{\rm{AF}}}}}}647},\cdots ,{y}_{i}^{{{{{{\rm{AF}}}}}}647},\cdots ,{y}_{N}^{{{{{{\rm{AF}}}}}}647}\right]}^{{{{{{\rm{T}}}}}}}$, ${{{{{{\bf{x}}}}}}}^{{{{{{\rm{JF}}}}}}549}={\left[{x}_{1}^{{{{{{\rm{JF}}}}}}549},{x}_{2}^{{{{{{\rm{JF}}}}}}549},\cdots ,{x}_{i}^{{{{{{\rm{JF}}}}}}549},\cdots ,{x}_{N}^{{{{{{\rm{JF}}}}}}549}\right]}^{{{{{{\rm{T}}}}}}}$, ${{{{{{\bf{y}}}}}}}^{{{{{{\rm{JF}}}}}}549}={\left[{y}_{1}^{{{{{{\rm{JF}}}}}}549},{y}_{2}^{{{{{{\rm{JF}}}}}}549},\cdots ,{y}_{i}^{{{{{{\rm{JF}}}}}}549},\cdots ,{y}_{N}^{{{{{{\rm{JF}}}}}}549}\right]}^{{{{{{\rm{T}}}}}}}$ ($\left[{x}_{i}^{{{{{{\rm{CHX}}}}}}},{y}_{i}^{{{{{{\rm{CHX}}}}}}}\right]$ denotes the $x,y$ coordinates of the $i$th bead in channel CHX) were submitted to a 2nd polynomial function (Eq. 1) to solve the mapping coefficient $\left[{{{{{\bf{k}}}}}}x,{{{{{\bf{k}}}}}}y\right]$,

1 $${{{{{\bf{A}}}}}}\left[\begin{array}{cc}{{{{{\bf{k}}}}}}x & {{{{{\bf{k}}}}}}y\end{array}\right]=\left[\begin{array}{cc}{{{{{{\bf{x}}}}}}}^{{{{{{\rm{AF}}}}}}647} & {{{{{{\boldsymbol{y}}}}}}}^{{{{{{\rm{AF}}}}}}647}\end{array}\right]$$

where ${A}_{{ij}}{{{{{\boldsymbol{=}}}}}}{\left({x}_{i}^{{{{{{\rm{JF}}}}}}549}\right)}^{\left\lfloor j/3\right\rfloor }{\left({y}_{i}^{{{{{{\rm{JF}}}}}}549}\right)}^{j-3* \left\lfloor j/3\right\rfloor }$ ($\left\lfloor x\right\rfloor$ denotes the maximum integer smaller than $x$); ${{{{{\bf{k}}}}}}x{{{{{\boldsymbol{=}}}}}}{\left[{{kx}}_{0},{{kx}}_{1},{{kx}}_{2},\cdots ,{{kx}}_{8}\right]}^{{{{{{\rm{T}}}}}}}$ and ${{{{{\bf{k}}}}}}y{{{{{\boldsymbol{=}}}}}}{\left[{{ky}}_{0},{{ky}}_{1},{{ky}}_{2},\cdots ,{{ky}}_{8}\right]}^{{{{{{\rm{T}}}}}}}$ are the 2nd polynomial mapping coefficient. This plastic wrapping method has been widely used for the correction of the chromatic aberrations in multi-color imaging^68^.

### Correlation functions for analysis of SMLM data

For Auto-Pair Correlation (PC) analyses, the list of the localizations of the examined molecules within the center region (~6 × 6 µm^2^) of each nucleus was submitted to calculate the spatial auto-correlation^68^. As mentioned above (in *SR microscopy* method section), localizations appearing in consecutive frames within 2.5 times of the average localization accuracy was considered as one localization^69^. The correlation was calculated as a function $g\left(r\right)$ of the pair-wise distance $r$, and fitted into a two Gaussian model as in Eq. (2):

2 $$g\left(r\right)=\frac{1}{4\pi {\sigma }^{2}\left\langle \rho \right\rangle }{{\exp }}\left(-\frac{{r}^{2}}{4{\sigma }^{2}}\right)+A{{\exp }}\left[-\frac{{r}^{2}}{4\left({\sigma }^{2}+{r}_{{{{{{\rm{app}}}}}}}^{2}\right)}\right]+1$$

where $\sigma$ and $\left\langle \rho \right\rangle$ are the localization precision and the average density of the examined molecules, respectively. The second term in Eq. (2) is the auto-correlation of the Gaussian modeled focus where $A$ is proportional to the average probability of finding molecules around each other and ${r}_{{{{{{\rm{app}}}}}}}$ is the apparent average radius of the focus. The average molecular content within each focus can then be derived as (3)

3 $$\left\langle N\right\rangle ={\iint }_{-{{\infty }}}^{+{{\infty }}}\left\langle \rho \right\rangle A\,{{\exp }}\left(-\frac{{x}^{2}+{y}^{2}}{2{r}_{{{{{\rm{app}}}}}}^{2}}\right){{{{{\rm{d}}}}}}x{{{{{\rm{d}}}}}}y=2\pi \left\langle \rho \right\rangle A{r}_{{{{{\rm{app}}}}}}^{2}$$

and the density of the foci was estimated by $\left\langle \rho \right\rangle /\left\langle N\right\rangle$.

For Cross-PC analyses, the list of the localizations of the two interested molecules within the same nucleus was submitted and the correlation function $c\left(r\right)$ was fitted into a symmetric Gaussian model (Eq. 4):

4 $$c\left(r\right)=A\left({{\exp }}\left[-\frac{{\left(r-{x}_{c}\right)}^{2}}{2{\sigma }^{2}}\right]+{{\exp }}\left[-\frac{{\left(r+{x}_{c}\right)}^{2}}{2{\sigma }^{2}}\right]\right)+1$$

where $A$ is the correlation amplitude; ${x}_{c}$ and $\sigma$ is the center-to-center correlation distance and the dispersion radius of the correlation, respectively. Given the mathematical structure of the correlation function ${C}_{{AB}}\left(d\right)$ between *P* and *Q* (Eq. 5):

5 $${C}_{{PQ}}\left(d\right)=\frac{{\left\langle {\rho }_{P}\left(R\right){\rho }_{Q}\left(R+d\right)\right\rangle }_{R}}{{\left\langle {\rho }_{P}\right\rangle }_{R}{\langle {\rho }_{Q}\rangle }_{R}}$$

we derive $A{\langle {\rho }_{Q}\rangle }_{R}$ to estimate the average local density of *Q* around each *P* and $A{\left\langle {\rho }_{P}\right\rangle }_{R}$ to estimate the average local density of *P* around each *Q*. Representative Pair- and Cross-Correlation data and fitted curves, and detailed description of Eq. (5) in Supplementary Note 1.

### Live-cell single-molecule tracking

U2OS cells expressing Cerulean-PCNA and siRNA-resistant HaloTag-USP1 fusions were transfected with or without USP1 siRNA for 72 h to knockdown endogenous USP1, and subsequently labeled for 15 min with 1 nM JF646 ligand prior to imaging on a custom-configured ASI microscope. Note that the concentration of JF646 ligand was optimized to a level that only a small fraction of USP1 molecules were fluorescently labeled for SMT. A 639 nm laser (ultralasers, MRL-FN-639-800) was expanded, collimated, and directed into an TIRF objective (Olympus, UApo N, 150× NA1.45) by a quad-edged dichroic beam splitter (Semrock, Di01-R405/488/532/635). Illumination was adjusted to the HILO mode and the illumination intensity was adjusted to balance between the signal-and-noise-ratio (SNR) and the photo-bleach of the JF646 fluorophore. A 405 nm laser (ultralasers, MDL-III-405-100) was used to mark the CFP-labeled PCNA as an S-phase indicator so that only PCNA-positive cells were selected for further analysis. JF646 and CFP were simultaneously illuminated and their signal was spectrally split (Di03-635 (Semrock) equipped in OptoSplit II (Teledyne Photometrics) and filtered (Semrock, FF01-676-37 and FF03-525-50, for JF646 and CFP, respectively) before collected by a sCMOS camera (Teledyne Photometrics, Prime 95B). Images were recorded on at 33 Hz (30 ms per frame) for 1000 frames. Raw image stacks were first averaged every 4 frames to eliminate the abundant free-diffusing USP1 molecules, and the background was subtracted with a rolling-ball algorithm (kernel radius is set to 10 pixels ~= 650 nm) via an ImageJ plugin^70^. The images were then submitted to the TrackMate ImageJ plugin^71^ for single-particle tracking. The durations of each track were collected for further analyses shown in Fig. 4.

### Cell proliferation and cytotoxicity assays

For cell proliferation assays, cells were seeded into 35 mm dishes at a density of 1 × 10^5^ cells per plate. At 24 h following plating, cells were treated with either siCtrl or siSPRTN siRNA, with or without 2.5 μM ML323. On day 3, cells were counted and re-seeded at 1 × 10^5^ cells per plate. On day 7, cells were again harvested and counted. Population doublings were calculated according to the following formula (ATCC): *n* = 3.32 (log UCY − log l) + *X*, where *n* = the final PDL number at end of a given subculture, UCY = the cell yield at that point, *l* = the cell number used as inoculum to begin that subculture, and *X* = the doubling level of the inoculum used to initiate the subculture being quantitated. For cisplatin cytotoxicity assay, cells were treated with either siCtrl or siSPRTN siRNAs for 24 h and then re-plated at a density of 5 × 10^3^ cells/well in 96-well plate format. The following day, cells were treated with the indicated doses of cisplatin and ML323 at a 1:4 ratio for 48 h. Cell viability was measured using the Cell-Titer Glo kit (Promega) according to the manufacturer’s instructions. The relative luminescence was measured using a Tecan Infinite 200 Pro plate reader. Results from cell proliferation and cytotoxicity assays were based on three independent experiments.

### Statistics and reproducibility

Primary data were recorded using Microsoft Excel and statistical analyses were performed using GraphPad Prism 8, FlowJo 10, ImageJ 1.52a, Matlab (v2017b), and OriginLab (2018) software. Exact p-values are provided for each experiment in the Source Data file associated with this article. A biological replicate dataset (using two CRISPR clones per cell line) was obtained for all Okseq samples. All SR experiments were performed at least in triplicate with >60 sample size, as listed in Table S1 in manuscript. All IF experiments, including DNA fiber analyses, are based on three biological replicates with a sample size *n* > 200. Western blotting experiments were performed in at least two independent experiments. Source data contains all raw data used for statistical analysis.

### Reporting summary

Further information on research design is available in the Nature Research Reporting Summary linked to this article.

## Supplementary information

Supplementary Information

Supplementary Movie 1

Supplementary Movie 2

Supplementary Movie 3

Supplementary Movie 4

Supplementary Movie 5

Supplementary Movie 6

Reporting Summary

## Data Availability

The data supporting the findings of this study are available from the corresponding authors upon reasonable request. The datasets generated during the current study are available in the NCBI GEO repository under accession number GSE175938. Source data for the figures and supplementary figures are provided as a Source Data file. Source data are provided with this paper.

## Source data

Source Data

## References

[1] Lecona E (2016). USP7 is a SUMO deubiquitinase essential for DNA replication. Nat. Struct. Mol. Biol..

[2] Povlsen LK (2012). Systems-wide analysis of ubiquitylation dynamics reveals a key role for PAF15 ubiquitylation in DNA-damage bypass. Nat. Cell Biol..

[3] Komander D, Rape M (2012). The ubiquitin code. Annu. Rev. Biochem..

[4] Reyes-Turcu FE, Ventii KH, Wilkinson KD (2009). Regulation and cellular roles of ubiquitin-specific deubiquitinating enzymes. Annu. Rev. Biochem..

[5] Nijman SMB (2005). A genomic and functional inventory of deubiquitinating enzymes. Cell.

[6] Pruneda, J. N. & Komander, D. Evaluating enzyme activities and structures of DUBs. *Methods Enzymol.* **618**, 321–341 (2019). 10.1016/bs.mie.2019.01.001 PMC7126802 30850058

[7] Luise C (2011). An Atlas of altered expression of deubiquitinating enzymes in human cancer. PLoS One.

[8] Huang TT (2006). Regulation of monoubiquitinated PCNA by DUB autocleavage. Nat. Cell Biol..

[9] Cohn MA (2007). A UAF1-containing multisubunit protein complex regulates the Fanconi anemia pathway. Mol. Cell.

[10] Maga G, Hübscher U (2003). Proliferating cell nuclear antigen (PCNA): A dancer with many partners. J. Cell Sci..

[11] Moldovan G-L, Pfander B, Jentsch S (2007). PCNA, the maestro of the replication fork. Cell.

[12] Chang DJ, Cimprich KA (2009). DNA damage tolerance: When it’s OK to make mistakes. Nat. Chem. Biol..

[13] Bienko M (2005). Ubiquitin-binding domains in Y-family polymerases regulate translesion synthesis. Science.

[14] Kannouche PL, Wing J, Lehmann AR (2004). Interaction of human DNA polymerase η with monoubiquitinated PCNA a possible mechanism for the polymerase switch in response to DNA damage. Mol. Cell.

[15] Watanabe K (2004). Rad18 guides polη to replication stalling sites through physical interaction and PCNA monoubiquitination. Embo J..

[16] Kim JM (2009). Inactivation of murine Usp1 results in genomic instability and a Fanconi anemia phenotype. Dev. Cell.

[17] Xu X (2019). Inhibition of ubiquitin specific protease 1 sensitizes colorectal cancer cells to DNA-damaging chemotherapeutics. Front. Oncol..

[18] Jones MJ, Colnaghi L, Huang TT (2012). Dysregulation of DNA polymerase κ recruitment to replication forks results in genomic instability. Embo J..

[19] Lim KS (2018). USP1 is required for replication fork protection in BRCA1-deficient tumors. Mol. Cell.

[20] Dungrawala H (2015). The replication checkpoint prevents two types of fork collapse without regulating replisome stability. Mol. Cell.

[21] García-Santisteban I, Peters GJ, Giovannetti E, Rodríguez JA (2013). USP1 deubiquitinase: Cellular functions, regulatory mechanisms, and emerging potential as target in cancer therapy. Mol. Cancer.

[22] Kee Y, Huang TT (2016). Role of deubiquitinating enzymes in DNA repair. Mol. Cell Biol..

[23] Lin H-C (2018). C-terminal end-directed protein elimination by CRL2 ubiquitin ligases. Mol. Cell.

[24] Piatkov KI, Colnaghi L, Békés M, Varshavsky A, Huang TT (2012). The auto-generated fragment of the Usp1 deubiquitylase is a physiological substrate of the N-end rule pathway. Mol. Cell.

[25] Conti C (2007). Replication fork velocities at adjacent replication origins are coordinately modified during DNA replication in human cells. Mol. Biol. Cell.

[26] Nieminuszczy J, Schwab RA, Niedzwiedz W (2016). The DNA fibre technique—tracking helicases at work. Methods.

[27] Ge XQ, Jackson DA, Blow JJ (2007). Dormant origins licensed by excess Mcm2–7 are required for human cells to survive replicative stress. Gene Dev..

[28] Ibarra A, Schwob E, Méndez J (2008). Excess MCM proteins protect human cells from replicative stress by licensing backup origins of replication. Proc. Natl Acad. Sci. USA.

[29] Mansilla SF (2016). Cyclin Kinase-independent role of p21CDKN1A in the promotion of nascent DNA elongation in unstressed cells. Elife.

[30] Lukas C (2011). 53BP1 nuclear bodies form around DNA lesions generated by mitotic transmission of chromosomes under replication stress. Nat. Cell Biol..

[31] Harrigan JA (2011). Replication stress induces 53BP1-containing OPT domains in G1 cells. J. Cell Biol..

[32] Chen Y-H (2019). Transcription shapes DNA replication initiation and termination in human cells. Nat. Struct. Mol. Biol..

[33] Petryk N (2016). Replication landscape of the human genome. Nat. Commun..

[34] McGuffee SR, Smith DJ, Whitehouse I (2013). Quantitative, genome-wide analysis of eukaryotic replication initiation and termination. Mol. Cell.

[35] Lui, S. K. L. et al. Monitoring genome-wide replication fork directionality by Okazaki fragment sequencing in mammalian cells. *Nat. Protoc.* **16**, 1–26 (2021). 10.1038/s41596-020-00454-5 PMC8792808 33442052

[36] Lee WTC (2021). Single-molecule imaging reveals replication fork coupled formation of G-quadruplex structures hinders local replication stress signaling. Nat. Commun..

[37] Whelan DR (2018). Spatiotemporal dynamics of homologous recombination repair at single collapsed replication forks. Nat. Commun..

[38] Tonzi P, Yin Y, Lee CWT, Rothenberg E, Huang TT (2018). Translesion polymerase kappa-dependent DNA synthesis underlies replication fork recovery. Elife.

[39] Chen Y-H (2015). ATR-mediated phosphorylation of FANCI regulates dormant origin firing in response to replication stress. Mol. Cell.

[40] Stingele J, Schwarz MS, Bloemeke N, Wolf PG, Jentsch S (2014). A DNA-dependent protease involved in DNA-protein crosslink repair. Cell.

[41] Vaz B (2016). Metalloprotease SPRTN/DVC1 orchestrates replication-coupled DNA-protein crosslink repair. Mol. Cell.

[42] Lessel D (2014). Mutations in SPRTN cause early onset hepatocellular carcinoma, genomic instability, and progeroid features. Nat. Genet..

[43] Lopez-Mosqueda J (2016). SPRTN is a mammalian DNA-binding metalloprotease that resolves DNA-protein crosslinks. Elife.

[44] Stingele J (2016). Mechanism and regulation of DNA-protein crosslink repair by the DNA-dependent metalloprotease SPRTN. Mol. Cell.

[45] Mórocz M (2017). DNA-dependent protease activity of human Spartan facilitates replication of DNA–protein crosslink-containing DNA. Nucleic Acids Res..

[46] Mosbech A (2012). DVC1 (C1orf124) is a DNA damage-targeting p97 adaptor that promotes ubiquitin-dependent responses to replication blocks. Nat. Struct. Mol. Biol..

[47] Maskey RS (2017). Spartan deficiency causes accumulation of Topoisomerase 1 cleavage complexes and tumorigenesis. Nucleic Acids Res..

[48] Maskey RS (2014). Spartan deficiency causes genomic instability and progeroid phenotypes. Nat. Commun..

[49] Juhasz S (2012). Characterization of human Spartan/C1orf124, an ubiquitin-PCNA interacting regulator of DNA damage tolerance. Nucleic Acids Res..

[50] Saha LK (2021). Replication-dependent cytotoxicity and Spartan-mediated repair of trapped PARP1–DNA complexes. Nucleic Acids Res..

[51] Halder S (2019). SPRTN protease and checkpoint kinase 1 cross-activation loop safeguards DNA replication. Nat. Commun..

[52] Maddi K (2020). Wss1 promotes replication stress tolerance by degrading histones. Cell Rep..

[53] Centore RC, Yazinski SA, Tse A, Zou L (2012). Spartan/C1orf124, a reader of PCNA ubiquitylation and a regulator of UV-induced DNA damage response. Mol. Cell.

[54] Mórocz, M. et al. DNA-dependent protease activity of human Spartan facilitates replication of DNA–protein crosslink-containing DNA. *Nucleic Acids Res.*10.1093/nar/gkw1315 (2017). 10.1093/nar/gkw1315 PMC5389635 28053116

[55] Chen J (2014). Single-molecule dynamics of enhanceosome assembly in embryonic stem cells. Cell.

[56] Liang Q (2014). A selective USP1–UAF1 inhibitor links deubiquitination to DNA damage responses. Nat. Chem. Biol..

[57] Zhao S (2020). A ubiquitin switch controls autocatalytic inactivation of the DNA–protein crosslink repair protease SPRTN. Nucleic Acids Res..

[58] Dharadhar, S., Dijk, W. J., Scheffers, S., Fish, A. & Sixma, T. K. Insert L1 is a central hub for allosteric regulation of USP1 activity. *Embo Rep.* **22**, e51749 (2021). 10.15252/embr.202051749 PMC8024992 33619839

[59] Rennie, M. L., Arkinson, C., Chaugule, V. K., Toth, R. & Walden, H. Structural basis of FANCD2 deubiquitination by USP1−UAF1. *Nat. Struct. Mol. Biol.* **28**, 1–9 (2021). 10.1038/s41594-021-00576-8 33795880

[60] Lange SM (2021). Deubiquitinases: From mechanisms to their inhibition by small molecules. Mol. Cell.

[61] Chaudhuri AR (2016). Replication fork stability confers chemoresistance in BRCA-deficient cells. Nature.

[62] Békés M (2016). Recognition of Lys48-linked Di-ubiquitin and deubiquitinating activities of the SARS coronavirus papain-like protease. Mol. Cell.

[63] Dungrawala H, Cortez D (2014). Purification of proteins on newly synthesized DNA using iPOND. Methods Mol. Biol..

[64] Chen S (2019). iASPP mediates p53 selectivity through a modular mechanism fine-tuning DNA recognition. Proc. Natl Acad. Sci. USA.

[65] Holden U, Kapanidis AN (2011). DAOSTORM: An algorithm for high- density super-resolution microscopy. Nat. Methods.

[66] Huang F (2013). Video-rate nanoscopy using sCMOS camera–specific single-molecule localization algorithms. Nat. Methods.

[67] Huang F, Schwartz SL, Byars JM, Lidke KA (2011). Simultaneous multiple-emitter fitting for single molecule super-resolution imaging. Biomed. Opt. Express.

[68] Yin Y, Lee WTC, Rothenberg E (2019). Ultrafast data mining of molecular assemblies in multiplexed high-density super-resolution images. Nat. Commun..

[69] Veatch SL (2012). Correlation functions quantify super-resolution images and estimate apparent clustering due to over-counting. PLoS One.

[70] Schindelin J (2012). Fiji: An open-source platform for biological-image analysis. Nat. Methods.

[71] Tinevez J-Y (2017). TrackMate: An open and extensible platform for single-particle tracking. Methods.

