# Supplementary material for: USP1-trapping lesions as a source of DNA replication stress and genomic instability
Source: Nat Commun. 2022 Apr 1;13:1740. doi: 10.1038/s41467-022-29369-3 (PMC8975806; doi:10.1038/s41467-022-29369-3)
Supplement: Supplementary file 1 — Supplementary Information [file 41467_2022_29369_MOESM1_ESM.pdf]

Supplementary Information

USP1-trapping lesions as a source of DNA replication stress and genomic instability

Kate E. Coleman[1,5], Yandong Yin[1,4,5], Sarah Kit Leng Lui[1], Sarah Keegan[1,2], David Fenyo[1,2], Duncan J. Smith[3], Eli Rothenberg[1], and Tony T. Huang[1]*

[1]Department of Biochemistry & Molecular Pharmacology, New York University School of Medicine, New York, NY, 10016, USA

[2]Institute for Systems Genetics, New York University School of Medicine, New York University School of Medicine, New York, NY, USA

[3]Department of Biology, New York University School of Medicine, New York, NY, 10016, USA

[4]present address: Institute of Chemical Biology, Shenzhen Bay Laboratory, School of Chemical Biology and Biotechnology, Peking University Shenzhen Graduate School, Shenzhen, China

[5]equal contribution

*Correspondence to: Tony.huang@nyumc.org

This File includes:

Supplementary Figures 1-7
Supplementary Movies 1-6
Supplementary Tables 1-2
Supplementary Note 1

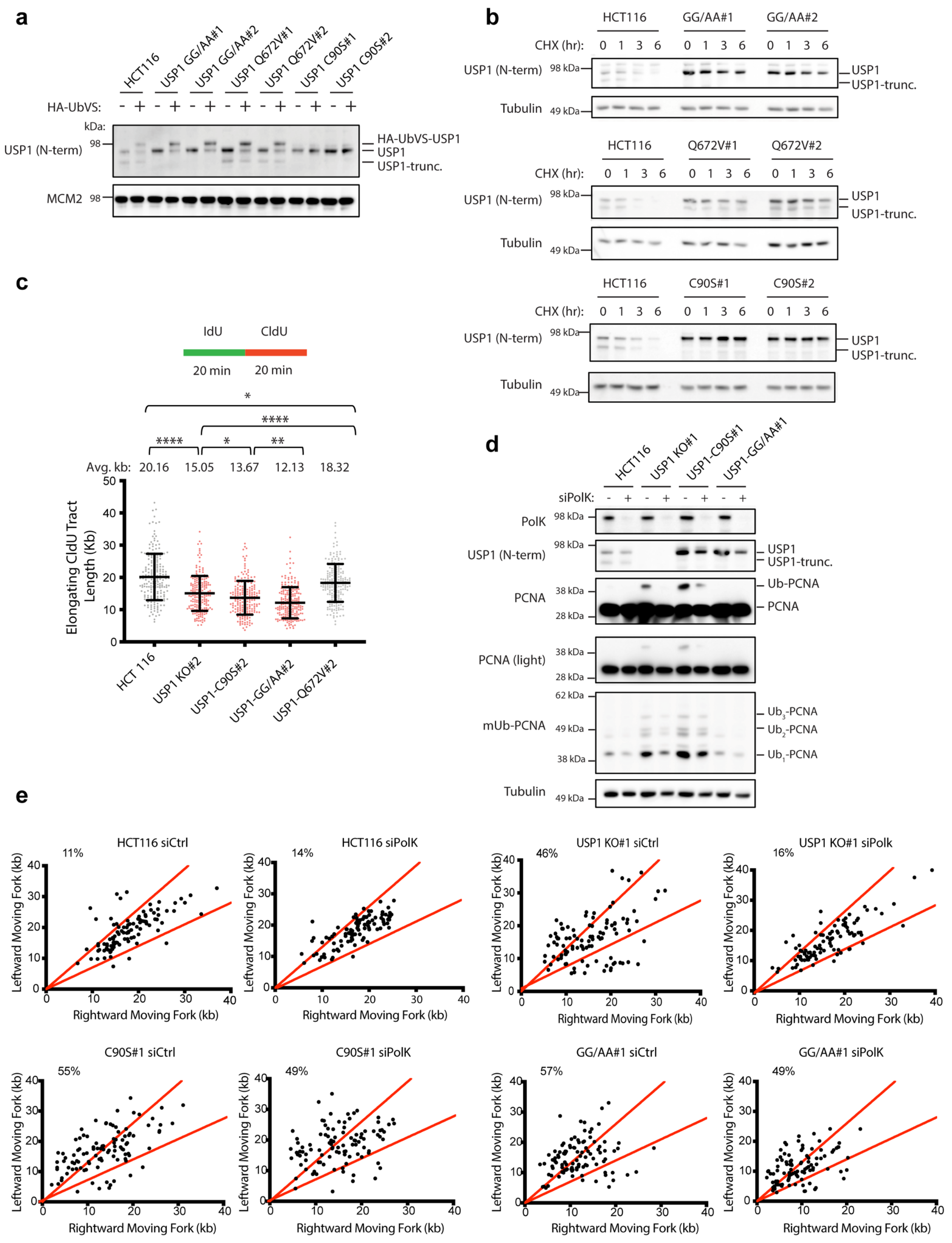

Supplementary Figure 1

Supplementary Figure 1. Characterization of HCT116 parental and USP1 knock-in mutant cell lines for defects in endogenous replication stress. **a**, Extracts of HCT116 parental and USP1 mutant cells were incubated with or without HA-Ub-VS DUB probe and analyzed by immunoblotting for USP1. MCM2 serves as a loading control. **b**, HCT116 cell lines were treated with 1 mM cycloheximide (CHX) for the indicated times to inhibit protein translation. USP1 levels were analyzed by immunoblotting. **c**, Alternative clones for each USP1 knockout or knock-in mutant cell line were pulse-labeled sequentially with IdU and CldU for 20 min each and subjected to DNA fiber analysis of elongating fork speed. CldU tract lengths of elongating forks are plotted with mean and -/+ SD for 3 independent experiments (n=200 elongating forks), and p values were calculated using the Mann-Whitney rank-sum t-test (ns=no significance, *p<0.05, **p<0.01, ****p<0.0001, two-tailed). **d**, HCT116 cell lines were treated with control or Polκ siRNA for 72 hours and analyzed by immunoblotting with the indicated antibodies. **e**, CldU tract lengths were measured for left and right forks emanating from the same origin of replication (n=100 bi-directional forks (origins)), as in Figure 1E. If the difference in fork length was more than 30% (outside of the red lines) they were classified as asymmetric forks. Percentages of asymmetric forks from each sample are indicated.

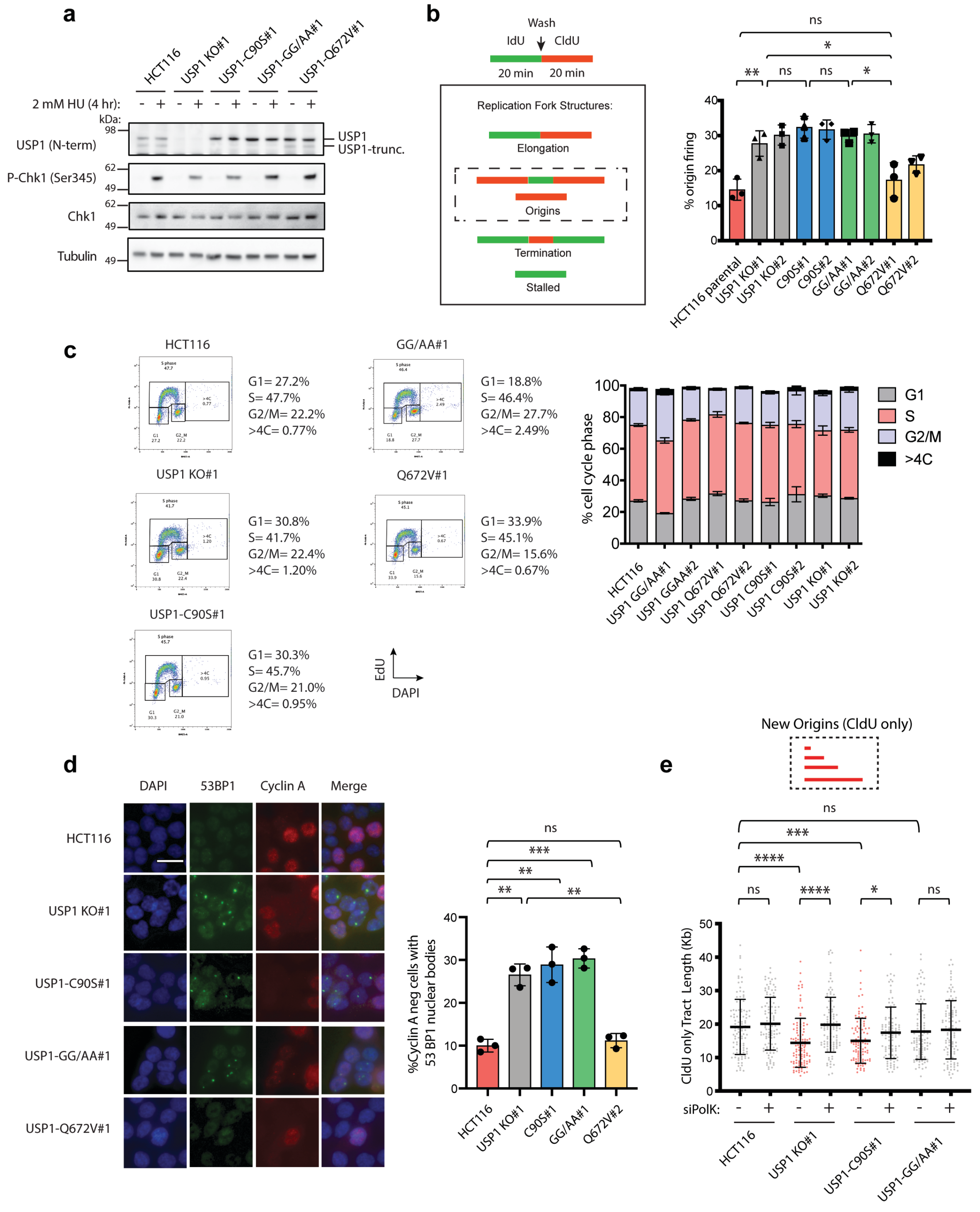

Supplementary Figure 2

Supplementary Figure 2. Cell cycle effects of USP1 autocleavage mutants. a, HCT116 cell lines were treated with or without 2 mM HU for 4 hours and subjected to immunoblotting analysis with the indicated antibodies. **b**, Schematics of different classes of replication structures from DNA fiber analysis used to determine the frequency of origins for each of the indicated HCT116 cell lines. The percentage of new origin firing was calculated as the sum of both bi-directional red-green-red tracts and red only tracks over the total number of replication structures. Data for % origin firing are represented by mean and -/+ SD for three independent experiments (n=200 events) and p-values were calculated using t-test with Welch's correction (ns=no significance, *p<0.05, **p<0.01, two-tailed). **c**, USP1 WT and mutant cells were pulse-labeled with 10 μM EdU for 30 min and analyzed by flow cytometry for DNA synthesis (EdU) and DAPI staining for DNA content (10,000 events per sample). Percentages of cells in G1, S, and G2/M phases are graphed for three biological replicates. Error bars indicate SEM. **d**, Asynchronously growing HCT116 cell lines were fixed and co-stained for Cyclin A and 53BP1 (counterstained with DAPI). Representative images for each cell line are shown. Cells that were Cyclin A negative with 53BP1 nuclear bodies were quantified and graphed with mean and -/+ SD for three independent experiments (n=300 Cyclin A negative cells per experiment). P-values were calculated using t-test with Welch's correction (ns=no significance, **p<0.01, ***p<0.001, two-tailed). Scale bar = 20 μM **e**, Cells were treated with either control or Polκ siRNA for 72 hours and subjected to DNA fiber analysis. Replication origins firing during the second labeling period (red only tracts) were analyzed for replication fork speed. CldU tract lengths of elongating forks are plotted with mean and -/+ SD for 3 independent experiments (n=100 red only origins), and p values were calculated using the Mann-Whitney rank-sum t-test (ns=no significance, *p<0.05, ***p<0.001, ***p<0.0001), two-tailed).

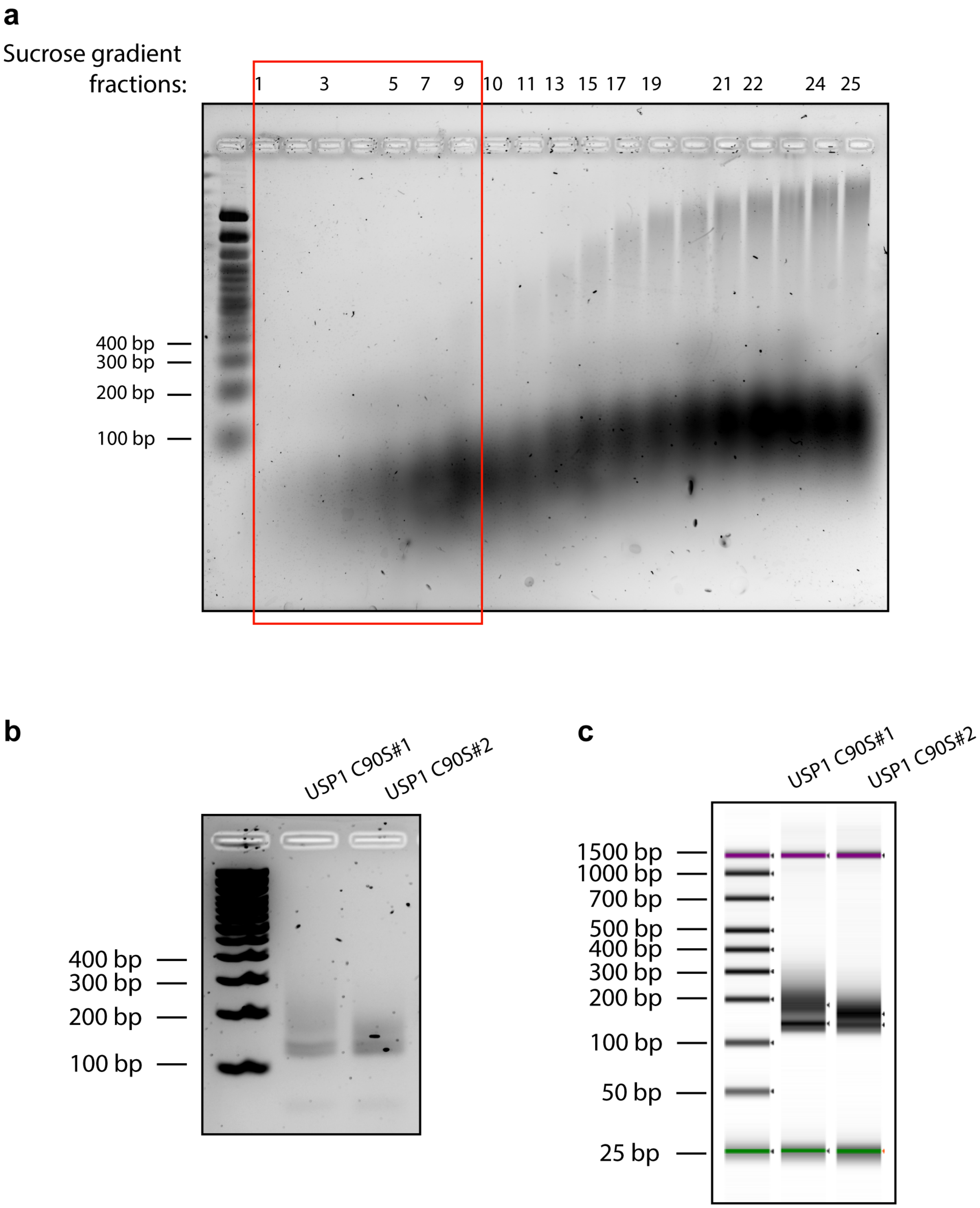

Supplementary Figure 3

Supplementary Figure 3. A representative sample of genomic DNA fractionation of HCT116 cells for Ok-seq analysis. **a**, Alkaline gel image showing sucrose gradient of HCT116 USP1C90S#1 genomic DNA. Fractions 1-9 (below >300bp) as indicated within the red box are concentrated for further processing to generate the adaptor-ligated Okazaki fragments library. **b**, Representative PCR gel image of amplified libraries of Okazaki fragments for HCT116 USP1C90S#1 and HCT116 USP1C90S#2 cell lines. **c**, Tapestation image of amplified libraries in (**b**) after removal of any adaptor-dimers and primer-dimers as part of quality control.

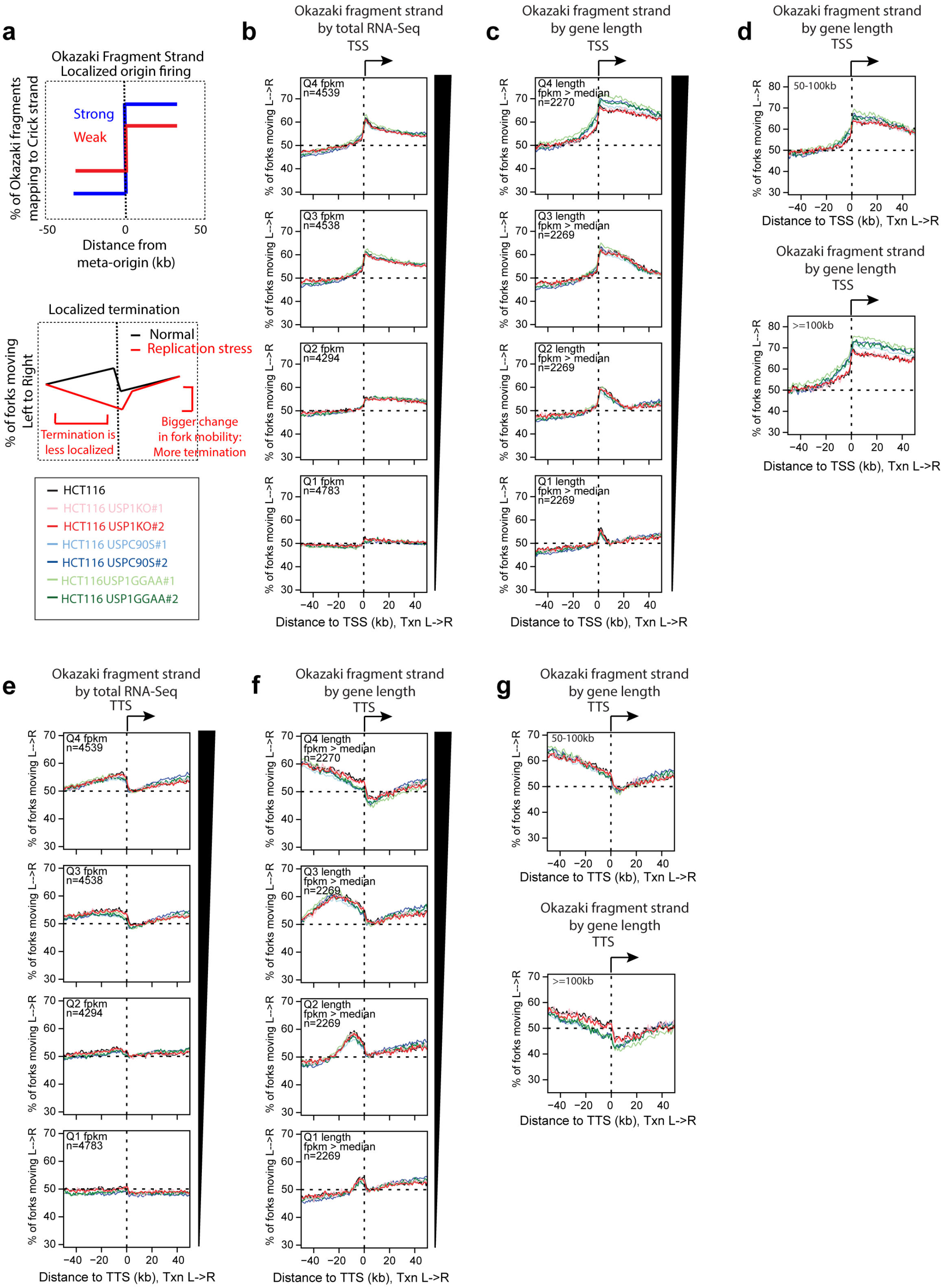

Supplementary Figure 4

Supplementary Figure 4. Alternative clones of USP1 mutant cell lines show similar delocalized patterns of replication initiation and termination within long genes. a, (upper panel) Schematic showing anticipated Okazaki fragment distributions around replication origins with strong (blue) versus weak (red) localized firing efficiencies. (lower panel) Schematic representation of Okazaki fragment distributions arising from replication termination at TTS under normal conditions (black) versus delocalized termination under replication stress (red). **b**, Percentage of replication forks moving left to right around TSS sorted by total RNA-seq read depth quartile (FPKM), (from ref[64]), for the indicated cell lines. P values were calculated using the Kruskal-Wallis test, using the regions 50-30kb upstream and 1-10kb downstream of the TSS. Effect sizes, shown as eff, were calculated using the same regions. The values were calculated comparing the USP1 mutant cell lines against the USP1 wild-type cell line. All statistics are presented in the Methods. **c**, Percentage of replication forks moving left to right around TSS of actively transcribed genes (FPKM>median), binned by gene length according to quartiles for transcribed genes. **d**, Same analysis as in (**c**), binned for long genes of 50-100 kb (upper panel) or >100 kb (lower panel). **e**, Percentage of replication forks moving left to right around TTS binned by RNA-seq read depth quartile for the indicated cell lines. **f**, Percentage of replication forks moving left to right around TTS of actively transcribed genes (FPKM>median), binned by gene length according to quartiles for transcribed genes. **g**, Same analysis as in (**f**), binned for long genes of 50-100 kb (upper panel) or >100 kb (lower panel).

a

b

c

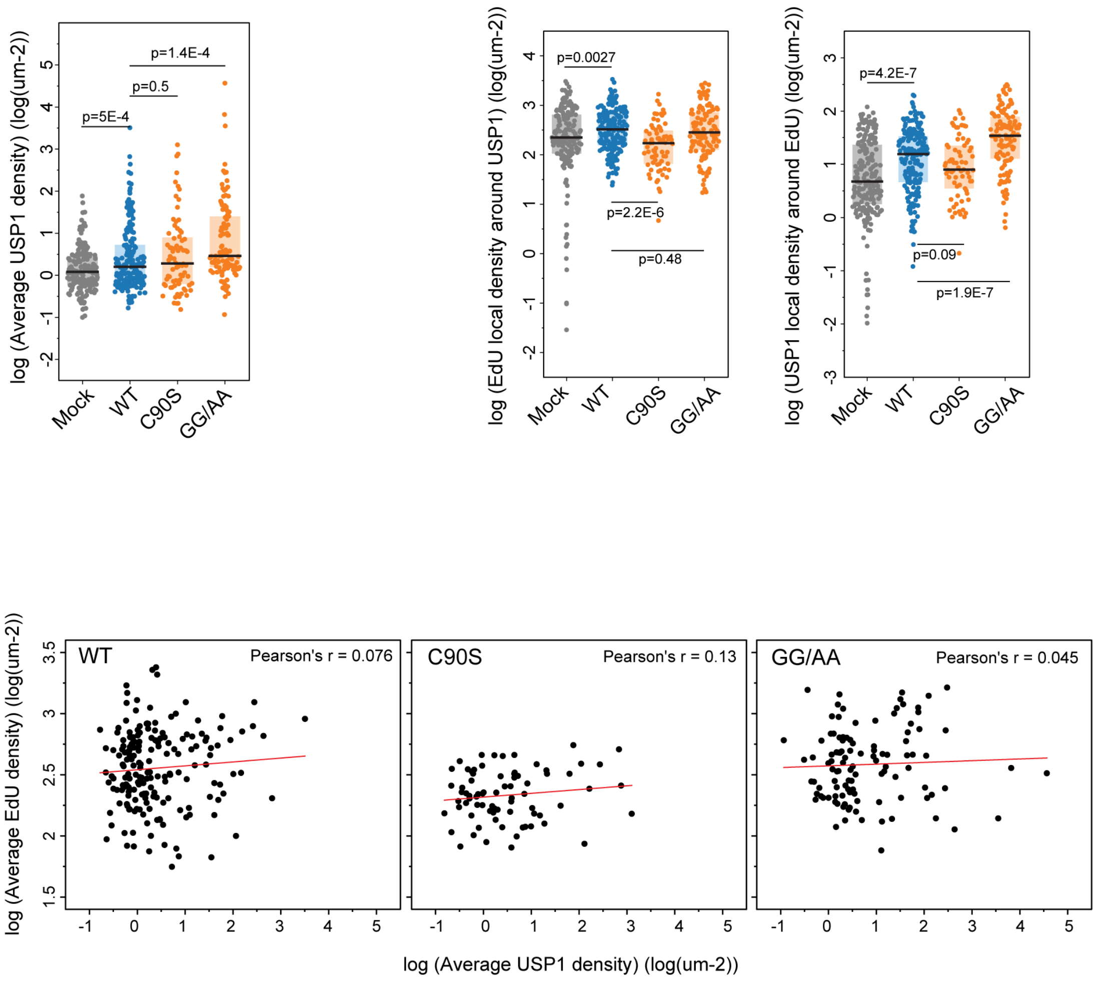

Supplementary Figure 5

Supplementary Figure 5. Incubation of Halotag ligand alone in Mock samples did not cause unspecific background localizations. a, Average USP1 density per nucleus. (mean -/+ std, n=166,183,76, 109 nuclei for Mock, USP1-WT, USP1-C90S, and USP1-GG/AA, respectively, p-values were calculated using the student's t-test). Boxes represent the 1st (25%) and 3rd (75%) percentile. **b**, Measurements of the average EdU density around USP1 (left), and the average USP1 density around EdU (right) are shown, based on at least two independent experiments (mean -/+ std, left: n= 183, 188, 71, 113 nuclei for Mock, WT, C90S, and GG/AA respectively; right n=207, 188, 71, 113 nuclei for Mock, WT, C90S and GG/AA respectively, p-values were calculated using the student's t-test). Boxes represent the 1st (25%) and 3rd (75%) percentile. **c**, Plots of the EdU density as a function of the USP1 density of the same nucleus (black dots, n = 183, 76, 109 nuclei for WT, C90S, and GG/AA, respectively). The linear regression (red line) is performed for all the samples, and no significant correlation is observed for all the samples.

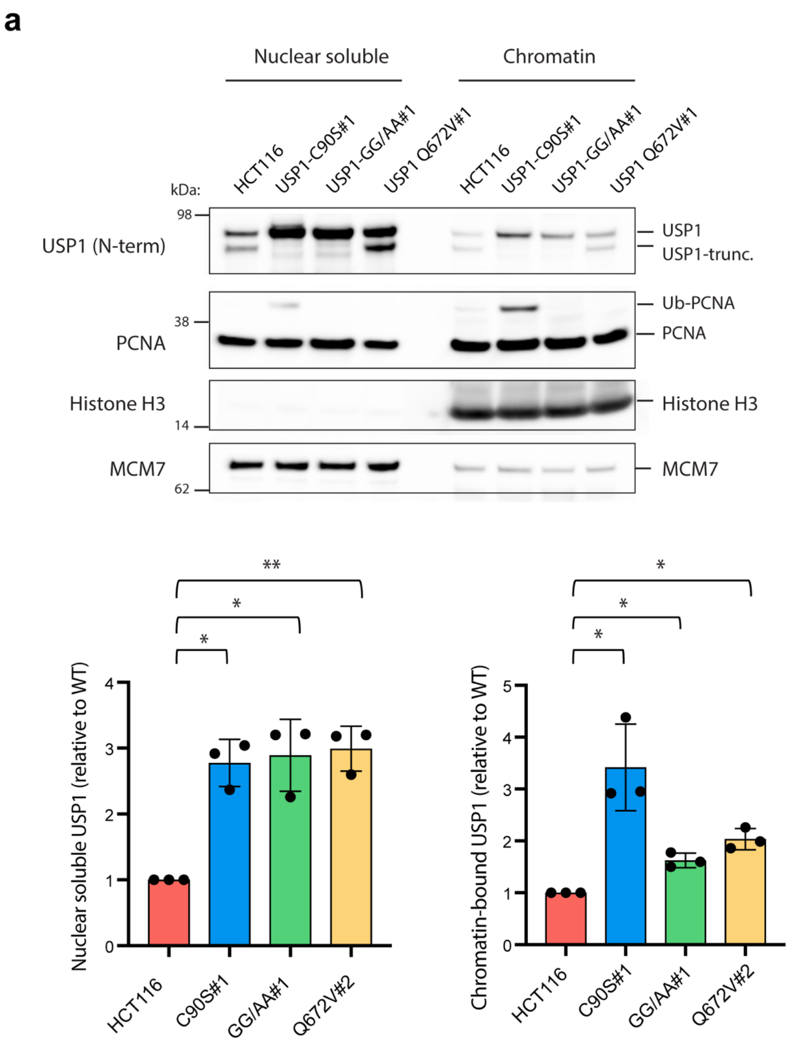

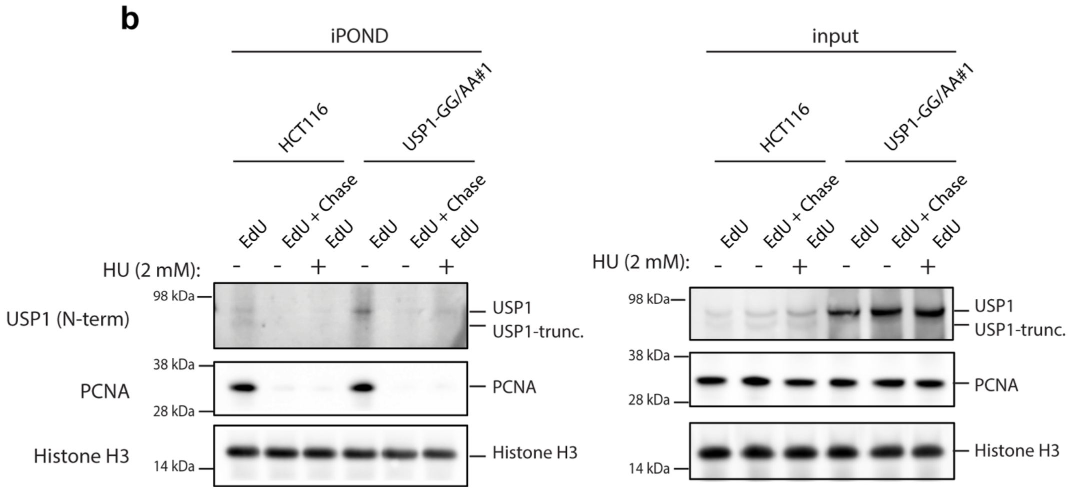

Supplementary Figure 6

Supplementary Figure 6. GG/AA mutant protein is enriched at replication forks by iPOND analysis. a, Immunoblotting analysis of nuclear soluble and chromatin-bound fractions prepared from USP1 WT and mutant cell lines. Histone H3 was used as a positive control marker for the chromatin fraction. Relative levels of USP1 (compared to WT=1) in the nuclear soluble and chromatin fractions were quantified by densitometry analysis. Mean with SD are plotted for 3 independent experiments and p-values were calculated using t-test with Welch's correction (*$p<0.05$, **$p<0.01$, two-tailed). **b**, HCT116 and USP1-GG/AA cells were pulse-labeled for 10 min with 10 μM EdU and then either collected directly (+EdU) or treated with 10 uM thymidine for 1 hr (+EdU + Chase) or 2 mM HU for 3 hours (+EdU +HU). iPOND and input samples were analyzed by immunoblotting with the indicated antibodies.

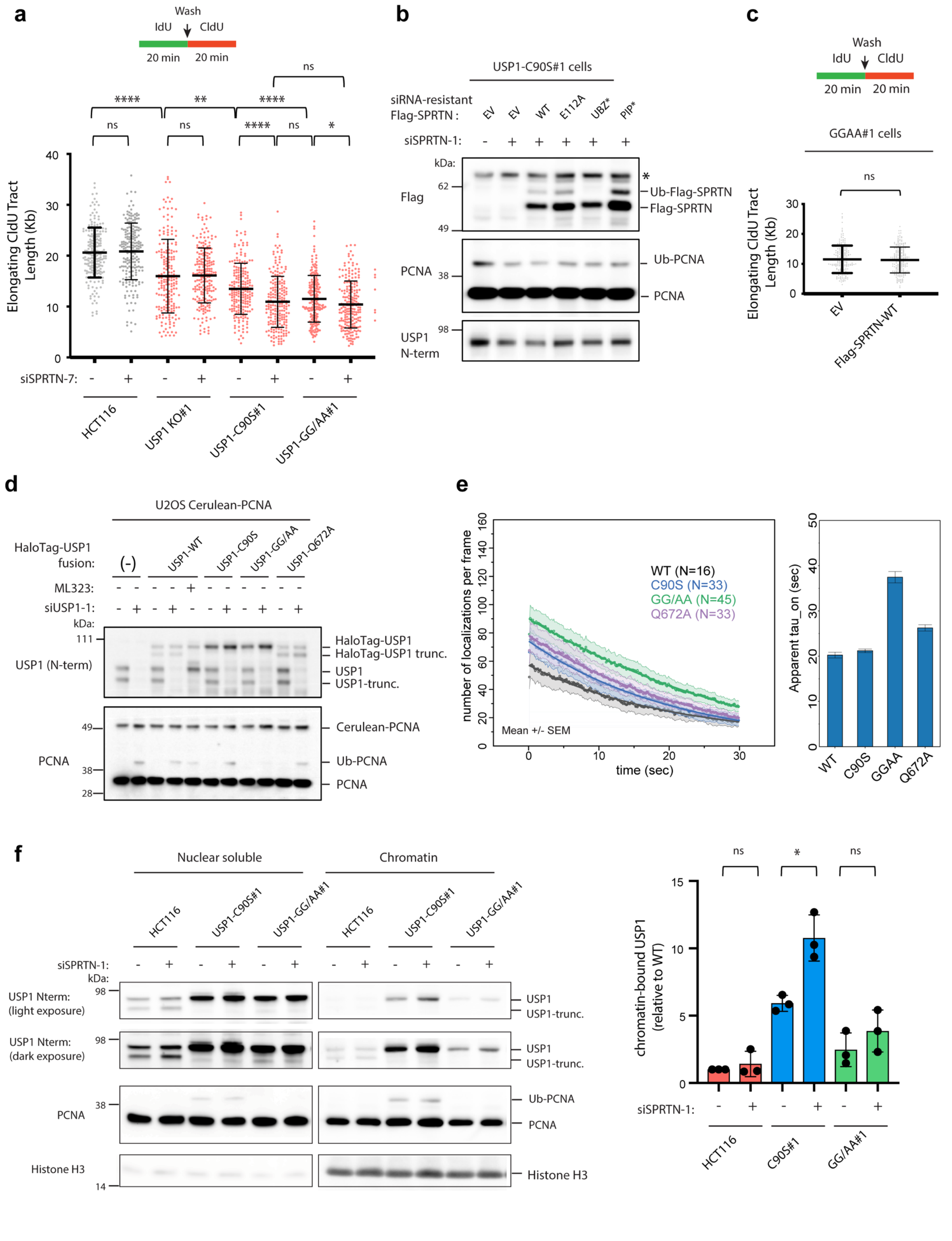

Supplementary Figure 7

Supplementary Figure 7. SPRTN depletion causes chromatin-enrichment of the USP1-C90S protein. a, HCT116 cell lines were treated with or without an alternative siRNA for SPRTN (siSPRTN-7) and subjected to DNA fiber analysis of fork speed, as previously described. CldU tract lengths of elongating forks are plotted with mean and -/+ SD for 3 independent experiments (n=200 elongating forks), and p values were calculated using the Mann-Whitney rank-sum t-test (ns=no significance, *p<0.05, **p<0.01, ****p<0.0001, two-tailed). **b**, HCT116 USP1-C90S cells were treated with either siCtrl or siSPRTN-1 siRNAs and complemented with siRNA-resistant Flag-SPRTN constructs prior to immunoblotting analysis for the indicated proteins. **c**, HCT116 USP1-GG/AA#1 cells overexpressing Flag-SPRTN-WT were compared to EV for measurements of elongating CldU Tract length. CldU tract lengths are plotted for 3 independent experiments (n=200 elongating forks) with mean -/+ SD indicated and p values were calculated using the Mann-Whitney rank-sum t-test. **d**, U2OS Cerulean-PCNA cells expressing siRNA-resistant HaloTag-USP1 fusion proteins were treated with or without siUSP1-1 and ML323 as indicated and subjected to immunoblotting analysis. **e**, (left graph) Average USP1 localizations per nucleus as a function of time (mean -/+ SEM, n= 16, 33, 45, and 33 nuclei for WT, C90S, GG/AA, andQ672A, respectively). Lines are the fitted single-exponential decay model. (right graph) The fitted bulk tau_on of different USP1 molecules as indicated. Mean -/+ std is the fitting result of the time course of tau_on in left graph. **f**, Cells were treated with or without siSPRTN-1 siRNA for 72 h and subjected to immunoblotting analysis of prepared nuclear soluble and chromatin fractions. Relative levels of chromatin-bound USP1 were quantified using densitometry analysis (normalized to WT=1) and the mean with SD are plotted for 3 independent experiments. P-values were calculated using t-test with Welch's correction (ns=no significance, *p<0.05, two-tailed).

Supplementary Movie 1 (see Source Files). Representative SR blinking movies for the same cell shown for AF647 for EdU signal as SR movie. Scale bar = 2000 nm. Only 500 frames are shown in order to reduce the file size.

Supplementary Movie 2 (see Source Files). Representative SR blinking movies for the same cell shown for JF549 for HaloTag USP1 signal as SR movie. Scale bar = 2000 nm. Only 500 frames are shown in order to reduce the file size.

Supplementary Movie 3 (see Source Files). **No significant HaloTag single-molecule signal for Mock-transfected cells.** Representative live-cell movie of U2OS cells using JF646-Halo ligand incubation without transfection of USP1 HaloTag constructs (Mock sample). Incubation time is same as for others. Left video panel is depicting the JF646 channel (USP1); Right video panel is CFP-PCNA as a marker for nucleus in S-phase.

Supplementary Movie 4-6 (see Source Files). Representative live-cell movies of U2OS cells stably expressing HaloTag-USP1 WT (Video 4), C90S (Video 5), and GG/AA (Video 6) fusion proteins.

Supplementary Table 1 – Key Resources Table

Antibodies

Reagent or Resource	Source	Identifier
USP1	Bethyl	A301-698A
MCM2	Bethyl	A300-191A
PCNA [PC10]	Abcam	ab29
Chk1	Abcam	ab2845
Histone H3	Abcam	ab1791
Ubiquityl PCNA (Lys 164)	Cell Signaling Technologies	13439S
Chk1 (Phospho Ser345)	Cell Signaling Technologies	2348S
α-Tubulin	CalBiochem	CP06
Polκ	Santa Cruz Biotechnology	sc-166667
MCM7	Santa Cruz Biotechnology	sc-9966
Flag [M2]	Sigma-Aldrich	F1804
Mouse anti-IdU [B44]	BD Biosciences	347580
rat anti-CldU [BU1/75 (ICR1)]	Abcam	ab6326
53BP1	Abcam	ab175933
Cyclin A2	Calbiochem	CC17
Goat anti-mouse IgG (H+L) Alexa Fluor 488	Thermo Fisher	A11001
Goat anti-rat IgG (H+L) Alexa Fluor 594	Thermo Fisher	A11007
Goat anti-mouse IgG (H+L) Alexa Fluor 546	Thermo Fisher	A11003
Goat anti-rabbit IgG Alexa Fluor 488	Thermo Fisher	A11008
Peroxidase AffiniPure Goat Anti-Mouse IgG (H+L)	Jackson Labs	115-035-003
Peroxidase AffiniPure Goat Anti-Rabbit IgG (H+L)	Jackson Labs	111-035-003

Chemicals, Peptides, and Recombinant Proteins

Reagent or Resource	Source	Identifier
HA-Ubiquitin-Vinyl Sulfone	Boston Biochem	U-212
USP1-UAF1 inhibitor, ML323	Calbiochem	531131
Cycloheximide	Calbiochem	239765
Biotin PEG-Azide	ThermoFisher Scientific	B10184
Biotin TEG-Azide	Berry & Associates	BT-1085
CuSO4 – click chemistry grade	Jena Biosciences	M1004-50
Ascorbic acid	Sigma-Aldrich	A92902-100G
Streptavidin, agarose conjugate beads	EMD Millipore	16-126
Dynabeads MyOne Streptavidin T1	ThermoFisher Scientific	65601
Hydroxyurea	Sigma-Aldrich	H8627
cOmplete Mini-Protease Inhibitor Cocktail	Roche	11836170001
5'-ethynyl-2deoxyuridine (EdU)	Sigma-Aldrich	900584
Thymidine (dT)	Sigma-Aldrich	T1895
5'-Iodo-2'deoxyuridine (IdU)	Sigma-Aldrich	I7125

5’-chloro-2’deoxyuridine (CldU)	Sigma-Aldrich	C6891
Janelia Fluor 549 HaloTag ligand	Promega	GA1110
Janelia Fluor 646 HaloTag ligand	Promega	GA1120
HaloTag TMRDirect Ligand	Promega	G2991
Cisplatin	EMD Millipore	232120-50MG
Lipofectamine 3000 transfection reagent	Thermo Fisher	L3000015
Lipofectamine RNAiMAX	Thermo Fisher	13778150
Opti-MEM	Gibco	31985-062
McCoy’s 5A Medium	Gibco	16600-082
DMEM	Gibco	11995-065
FBS	R&D Systems	S11150H
Prolong Gold Antifade Reagent	Cell Signaling Technologies	9071S
Paraformaldehyde 16% solution	Electron Microscopy Sciences	15710
Phenol:chloroform:isoamyl alcohol	Thermo Fisher	15593049
T4 DNA ligase	Enzymatics	L6030-LC-L
T4 polynucleotide kinase	New England Biolabs	M0201L
Proteinase K	Roche	3115828001

Critical Commercial Assays/Kits

Reagent or Resource	Source	Identifier
CellTiter-Glo Luminescent Cell Viability Assay	Promega	G7570
Click-iT PLUS EdU Imaging Kit, Alexa Fluor 647 dye	Thermo Fisher	C10640
Click-iT Plus EdU Alexa Fluor 594 Flow Cytometry assay kit	Thermo Fisher	C10646
Subcellular Protein Fractionation Kit for Cultured Cells	Thermo Fisher	78840

Experimental Models – Cell Lines

Reagent or Resource	Source	Identifier
HCT116	ATCC	
HEK293T	ATCC	
U2OS	ATCC	
HCT116 USP KO #1	This Study	Applied Stem Cell
HCT116 USP1 KO #2	This Study	Applied Stem Cell
HCT116 USP1-C90S #1	This Study	Applied Stem Cell
HCT116 USP1-C90S #2	This Study	Applied Stem Cell
HCT116 USP1-GG/AA #1	This Study	Applied Stem Cell
HCT116 USP1-GG/AA #2	This Study	Applied Stem Cell
HCT116 USP1-Q672V #1	This Study	Applied Stem Cell
HCT116 USP1-Q672V #2	This Study	Applied Stem Cell

U2OS NLS-mCerulean-PCNA		Gift from Huijun Xue (E. Rothenberg Lab); See Materials & Methods
U2OS HaloTag-USP1-WT (siRes) + NLS-mCerulean-PCNA	This Study	See Materials & Methods
U2OS HaloTag-USP1-C90S (siRes) + NLS-mCerulean-PCNA (siRes)	This Study	See Materials & Methods
U2OS HaloTag-USP1-GGAA (siRes) + NLS-mCerulean-PCNA	This Study	See Materials & Methods
U2OS HaloTag-USP1-Q672A (siRes) + NLS-mCerulean-PCNA	This Study	See Materials & Methods

Oligonucleotides

Reagent or Resource	Source	Sequence
All-Star Negative Control siRNA	Qiagen	Proprietary
PolK siRNA (siPolK-5)	Qiagen	5'-TGGAATTAGAACAAAGCCGAA-3
USP1 siRNA (siUSP1-1)	Qiagen	5'-TCGGCAATACTTGCTATCTTA-3'
SPRTN siRNA (siSPRTN-1; siSPRTN-7)	Qiagen	#1, 5'-AGCCAATATAACGGTATACCA-3',<br>#7, 5'- ACAGTTGGCAACATCCCTAAA-3'
USP1 siRNA-resistant-F primer	IDT	5'-GTGGGACTGAATAATCTTGGAAACACGTGTTACTT GAATAGTATACTTCAGG-3'
USP1 siRNA-resistant-R primer	IDT	5'-CCTGAAGTATACTATTCAAGTAACACGT GTTTCCAAGATTATTCAGTCCCA-3'
SPRTN siRNA-resistant-F primer	IDT	5'- CATCAACAGCCTGACTGGAGCAAACATCA CCGTGTACCATACTTTTCACGATG-3'
SPRTN siRNA-resistant-R primer	IDT	5'-CATCGTGAAAAGTATGGTACACGGTGATG TTTGCTCCAGTCAGGCTGTTGATG-3'
SPRTN_UBZ_F primer	IDT	5'-AGCAAAATGGTTAATGGCCCAGTTG GTCAGAATGAAGTTCTG-3'
SPRTN_PIPm_F primer	IDT	5'-AATGTTCTAAGCAACGCCGCTCCT AGAGTATCATTTG-3'
SPRTN_PIPm_R primer	IDT	5'-CAAATGATACTCTAGGAGCGGCGTTG CTTAGAACATT-3'

Recombinant DNA

Reagent or Resource	Source	Identifier
pHTn-HaloTag (EV)	Promega	G7721
pHTn-HaloTag-USP1-WT	This Study	See Materials & Methods

pHTn-HaloTag-USP1-C90S	This Study	See Materials & Methods
pHTn-HaloTag-USP1-GG/AA	This Study	See Materials & Methods
pHTn-HaloTag-USP1-Q672A	This Study	See Materials & Methods
pBabe_puro_HaloTag (EV)		Gift from Huijun Xue (E. Rothenberg lab); see materials & methods
pBabe puro HaloTag-USP1-WT (siRes)	This Study	See Materials & Methods
pBabe puro HaloTag-USP1-C90S (siRes)	This Study	See Materials & Methods
pBabe puro HaloTag-USP1-GGAA (siRes)	This Study	See Materials & Methods
pBabe puro HaloTag-USP1-Q672A (siRes)	This Study	See Materials & Methods
pcDNA3-Flag-SPRTN-WT	Addgene	110214
pcDNA3-Flag-SPRTN-E112A	Addgene	110215
pcDNA3-Flag-SPRTN-WT (siRes)	This Study	See Materials & Methods
pcDNA3-Flag-SPRTN-E112A (siRes)	This Study	See Materials & Methods
pcDNA3-Flag-SPRTN-UBZ* (siRes)	This Study	See Materials & Methods
pcDNA3-Flag-SPRTN-PIP* (siRes)	This Study	See Materials & Methods
pBabe-puro-NLS-mCerulean-PCNA		Gift from Gergely Rona (M. Pagano lab)

gRNA sequences for generation of CRISPR knock-in cell lines

gRNA name	Sequence (5'→3')	Notes
hUSP1_g1	TTTGTGGGACTGAATAATCT	Used to generate C90S mutation
hUSP1_g2	CTATCTTAATAGTATACTTC	Used to generate C90S mutation
hUSP1_g3	GAAGCTATTGGACTTCTTGG	Used to generate GG670-671AA and Q672V mutations
hUSP1_g4	GTAGAAGCTATTGGACTTCT	Used to generate GG670-671AA and Q672V mutations

Single-stranded deoxynucleotide donors for generation of CRISPR knock-in cells

Name	Sequence (5'→3')
hUSP1-C1539_GG670-671AA.g3.ssODN	GGTTGAGAATTATAATGATGAAGAAGTGTCAATTAGAGTTGGTGGAAATA CACAGCCAAGTAAAGTTTTGAACAAAAAAAAATGTA CAAAAGAGCAAAGCAGATTATGAGCTATACAACAAAGCCTC TAATCCTGATAAGGTTGCTAGTACAGCGTTTGCTGAAAATAGAAATTCT

hUSP1-C1540_C90S.g1.ssODN	ACATTAAATGCTACATCAATCTTTGCCTACTAAAGCTTTATGATATAGCA AAATTGTCAATTTACCTGAAGTATACTATTAAGATA AGTATTG TGGTAACAAGTTTTCTCTCTTCTCACAGTTT ATAGGTGAAGACTGTGCTGCAGGAACAACTTGATCACTATGAAATATAG
hUSP1-C1541_Q672V.g3.ssODN	TGTGGTTGAGAATTATAATGATGAAGAAGTGTCAATTAGAGTTGGTGGAA ATACACAGCCAAGTAAAGTTTTGAACAAAAAAAAATGTA AAAGAGCAAAGCAGATTATGAGCTATACAACAAAGC CTCTAATCCTGATAAGGTTGCTAGTACAGCGTTTGCTGAAAATAGAAATT CTGA

Software and Algorithms

Software	Source	
ImageJ v1.52a	https://imagej.nih.gov/ij/	
FlowJo v10	https://www.flowjo.com/	
GraphPad Prism v8	https://www.graphpad.com	
MatLab (v2017b)	https://www.mathworks.com/	
OriginLab (2018)	https://www.originlab.com/	

Supplementary Note 1. Representative Pair- and Cross-Correlation Data and Fitted Curves.

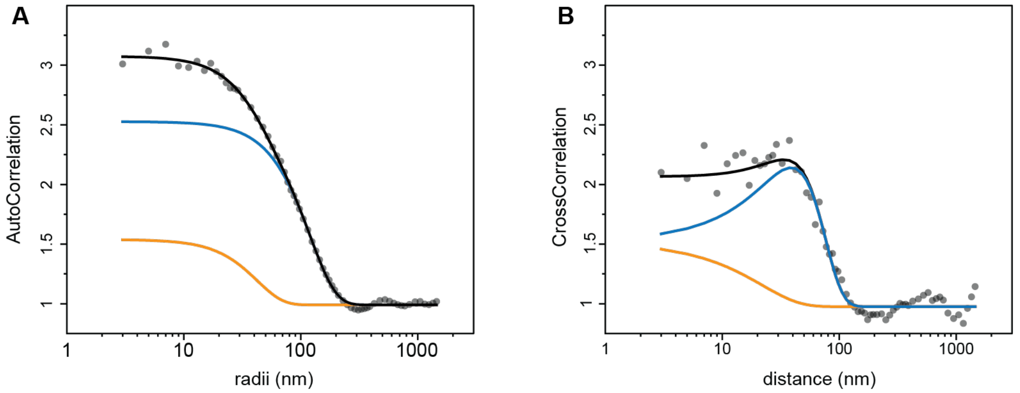

Auto- (A) and Cross- (B) correlation fitting examples. The auto correlation was fitted to Equation 5 and the cross correlation was fitted to a symmetric-gauss model as explained below.

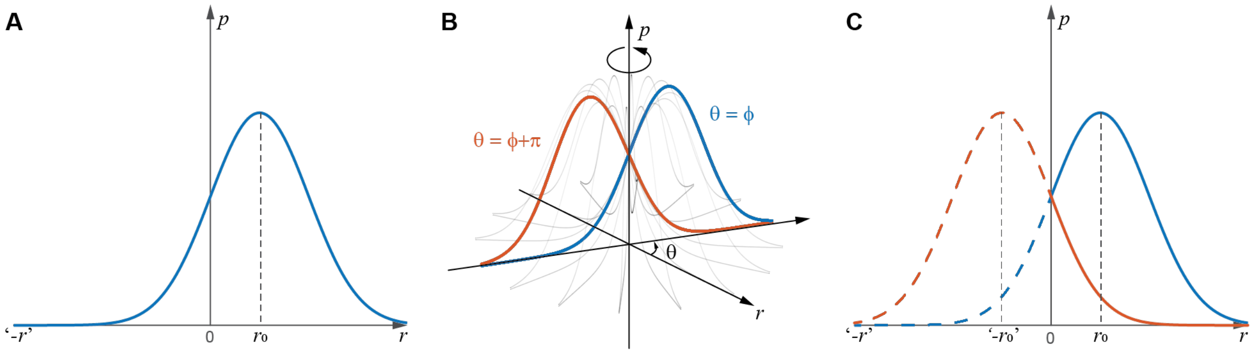

Considering the cross correlation between a P-Q pair, for each P, the Qs distribute at r_0 around P with some variance. Note that such probability distribution might expand to the '$-r$' axis due to the relatively large variance (A). Because the dumbbell-like P-Q pairs are randomly orientated in the nucleus, such distribution should identical from $\theta = 0 \sim 2\pi$. Note that the distribution at $\theta = \phi + \pi$ may expand from the '$-r$' axis to the r axis (B). Therefore, after averaging from 0 to 2π along θ, the final radial function (cross-correlation as a function of distance) is contributed by the symmetric distribution along r, shown in C as solid parts in both blue and orange colors.
In our data, a symmetric Gauss model perfectly approximates the final radial cross-correlation profile.

$$C(r) = A\exp\left[-\frac{(r-r_0)^2}{2\sigma^2}\right] + A\exp\left[-\frac{(r+r_0)^2}{2\sigma^2}\right]$$

Description of Equation 5 (how cluster densities are calculated). Equation 5 is the definition of the cross-correlation function $C_{\mathrm{PQ}}(d)$ (where equation 4 is its analytical model). More rigorously, it should be written in the vector format

$$C_{\mathrm{PQ}}(\mathbf{d}) = \frac{\langle \rho_{\mathrm{P}}(\mathbf{R})\rho_{\mathrm{Q}}(\mathbf{R}+\mathbf{d})\rangle_{\mathbf{R}}}{\langle \rho_{\mathrm{P}}(\mathbf{R})\rangle_{\mathbf{R}}\langle \rho_{\mathrm{Q}}(\mathbf{R})\rangle_{\mathbf{R}}}$$

Where $\rho_{\mathrm{P/Q}}(\mathbf{R})$ is the local density of species P/Q at a given location $\mathbf{R}$ in the image; $\langle\cdots\rangle_{\mathbf{R}}$ denotes the average of all the locations $\mathbf{R}$ across the image. As the P-Q pairs in the nucleus are randomly oriented, the 2D $C_{\mathrm{PQ}}(\mathbf{d}) = C_{\mathrm{PQ}}(d, \theta)$ is integrated across θ from 0 to 2π, resulting in the 1D radial distribution function $C_{\mathrm{PQ}}(d)$.

$\langle\rho_{\mathrm{P}}(\mathbf{R})\rho_{\mathrm{Q}}(\mathbf{R}+\mathbf{d})\rangle_{\mathbf{R}}$ is the pairing density[2] at distance $\mathbf{d}$, and thus $\langle\rho_{\mathrm{P}}(\mathbf{R})\rho_{\mathrm{Q}}(\mathbf{R}+\mathbf{d})\rangle_{\mathbf{R}}/\langle\rho_{\mathrm{P}}(\mathbf{R})\rangle_{\mathbf{R}}$, or equivalently $C_{\mathrm{PQ}}(\mathbf{d})\langle\rho_{\mathrm{Q}}(\mathbf{R})\rangle_{\mathbf{R}}$, denotes the average local density of Q that pairs with each P at distance $\mathbf{d}$, and vice versa. The amplitude, A, of the cross-correlation (equation 4) is the $C(d = r_0)$ where the cross-correlation reaches the maximum, and $A\langle\rho_{\mathrm{Q}}(\mathbf{R})\rangle_{\mathbf{R}}$ is used to estimate the average local density of Q pairing with each P. Note that if P and Q are randomly distributed (no cross-correlation signal) around each other, the local pairing density is 0. This distinguishes from direct counting the local density around each other, which would result in high local surrounding density when P and Q are densely but randomly distributed around each other.
