# Supplementary material for: USP1-trapping lesions as a source of DNA replication stress and genomic instability
Source: Nat Commun. 2022 Apr 1;13:1740. doi: 10.1038/s41467-022-29369-3 (PMC8975806; doi:10.1038/s41467-022-29369-3)
Supplement: Supplementary file 8 — Reporting Summary [file 41467_2022_29369_MOESM8_ESM.pdf]

nature portfolio

Corresponding author(s): Tony T. Huang

Last updated by author(s): Feb 18, 2022

Reporting Summary

Nature Portfolio wishes to improve the reproducibility of the work that we publish. This form provides structure for consistency and transparency in reporting. For further information on Nature Portfolio policies, see our Editorial Policies and the Editorial Policy Checklist.

Statistics

For all statistical analyses, confirm that the following items are present in the figure legend, table legend, main text, or Methods section.

n/a	Confirmed	
☐	☒	The exact sample size (*n*) for each experimental group/condition, given as a discrete number and unit of measurement
☐	☒	A statement on whether measurements were taken from distinct samples or whether the same sample was measured repeatedly
☐	☒	The statistical test(s) used AND whether they are one- or two-sided<br>*Only common tests should be described solely by name; describe more complex techniques in the Methods section.*
☒	☐	A description of all covariates tested
☐	☒	A description of any assumptions or corrections, such as tests of normality and adjustment for multiple comparisons
☐	☒	A full description of the statistical parameters including central tendency (e.g. means) or other basic estimates (e.g. regression coefficient) AND variation (e.g. standard deviation) or associated estimates of uncertainty (e.g. confidence intervals)
☐	☒	For null hypothesis testing, the test statistic (e.g. *F*, *t*, *r*) with confidence intervals, effect sizes, degrees of freedom and *P* value noted<br>*Give P values as exact values whenever suitable.*
☒	☐	For Bayesian analysis, information on the choice of priors and Markov chain Monte Carlo settings
☒	☐	For hierarchical and complex designs, identification of the appropriate level for tests and full reporting of outcomes
☐	☒	Estimates of effect sizes (e.g. Cohen's *d*, Pearson's *r*), indicating how they were calculated

Our web collection on statistics for biologists contains articles on many of the points above.

Software and code

Policy information about availability of computer code

Data collection	Super-resolution microscopy data were acquired using Micro-Manger (v 1.4).<br>Please see Methods section for detailed data acquisition description.

Data analysis	Data were aligned to hg19 using bowtie2, and analyzed using in-house python and R scripts<br><br>Super-resolution image reconstruction were performed through C++ (via Intel Core i7 7800X) and CUDA8.0 (via NVIDIA GTX 1060) using the Maximum Likelihood Estimation (MLE) algorithm.<br><br>Data presentation/graphing were performed mainly through Matlab (v2017b), Originlab(2018), and GraphPad Prism (v8).<br>Please see Methods section for detailed data analysis description.<br>Codes for Auto- and Triple-Correlation algorithms, as well as a testing demo (with simulation codes) are available at https://github.com/yiny02/direct-Triple-Correlation-Algorithm. The code is for Research and Educational Purposes for Non-Profit Academic and/or Research Institutions.<br><br>Software for data analysis:<br>ImageJ 1.52a<br>FlowJo v10<br>GraphPad Prism (v8)<br>Matlab (v2017b)<br>OriginLab (2018)

For manuscripts utilizing custom algorithms or software that are central to the research but not yet described in published literature, software must be made available to editors and reviewers. We strongly encourage code deposition in a community repository (e.g. GitHub). See the Nature Portfolio guidelines for submitting code & software for further information.

Data

Policy information about availability of data

All manuscripts must include a data availability statement. This statement should provide the following information, where applicable:

- Accession codes, unique identifiers, or web links for publicly available datasets
- A description of any restrictions on data availability
- For clinical datasets or third party data, please ensure that the statement adheres to our policy

All imaging and single-molecule data constitute a sizable dataset (>10TB) that cannot be reasonably maintained online. Raw data will be made available by the corresponding author upon request.

Data, including raw sequencing reads and tables used to generate source data for graphs in Figure 2 and Supplemental Figure 4 will be publicly available under GEO accession GSE175938. Custom scripts are available upon request from the corresponding author.

Field-specific reporting

Please select the one below that is the best fit for your research. If you are not sure, read the appropriate sections before making your selection.

☒ Life sciences ☐ Behavioural & social sciences ☐ Ecological, evolutionary & environmental sciences

For a reference copy of the document with all sections, see nature.com/documents/nr-reporting-summary-flat.pdf

Life sciences study design

All studies must disclose on these points even when the disclosure is negative.

Sample size	For all experiments sample size was not predetermined, as much data as possible was collected depending on the nature of the experiments or in order to perform proper statistical analysis.
Data exclusions	For all imaging experiments, EDU- and/or PCNA-positive (as pre-established S-phase markers) nuclei were selected for analysis, which were then analyzed equally. Details of nucleus selection were described in the manuscript.<br>No other data exclusion were performed.
Replication	A biological replicate dataset (using two CRISPR clones per cell line) was obtained for all Okseq samples.<br>All SR experiments were performed at least in triplicate with >60 sample size, as listed in Table S1 in manuscript.<br>Western blotting experiments were performed in at least two independent experiments.<br>IF experiment was performed in three independent experiments.<br>We followed the same protocols to generate replicates for each of our experiments, and the analysis of the data were reliably reproduced.
Randomization	For all imaging experiments, nuclei on coverslips were randomly selected for imaging.<br>For all single-molecule experiments, illuminated molecules were randomly selected for imaging.<br>For western blot experiment, no randomization is required as a proper internal control is present.
Blinding	For all data and imaging experiments, blinding was not possible as experimental conditions were evident from the image data. Image processing and analysis were done using computational pipelines that were applied equally to all conditions and replicates, therefore do not require blinding.

Reporting for specific materials, systems and methods

We require information from authors about some types of materials, experimental systems and methods used in many studies. Here, indicate whether each material, system or method listed is relevant to your study. If you are not sure if a list item applies to your research, read the appropriate section before selecting a response.

Materials & experimental systems

n/a	Involved in the study
☐	☒ Antibodies
☐	☒ Eukaryotic cell lines
☒	☐ Palaeontology and archaeology
☒	☐ Animals and other organisms
☒	☐ Human research participants
☒	☐ Clinical data
☒	☐ Dual use research of concern

Methods

n/a	Involved in the study
☒	☐ ChIP-seq
☒	☐ Flow cytometry
☒	☐ MRI-based neuroimaging

Antibodies

Antibodies used

mouse anti-IdU [B44] (BD Biosciences 347580, 1:150 dilution for DNA fiber assay)
rat anti-CldU [BU1/75 (ICR1)] (ab6326, Abcam ab6326, 1:200 dilution for DNA fiber assay)
USP1 (Bethyl A301-698A, 1:3000 dilution for WB)
MCM2 (Bethyl A300-191A, 1:10,000 dilution for WB)
MCM7 (Santa Cruz Biotechnology sc-9966, 1:5000 dilution for WB)
PCNA [PC10] (Abcam ab29, 1:3000 dilution for WB)
Chk1 (Abcam ab2845, 1:1000 dilution for WB)
Histone H3 (Abcam ab1791, 1:10,000 dilution for WB)
Ubiquityl-PCNA [D5C7P] (Cell Signaling Technologies 13439S, 1:1000 dilution for WB)
pChk1 (Phospho-Ser345) [133D3] (Cell Signaling Technologies 2348S, 1:5000 dilution for WB)
alpha-Tubulin [DM1A] (CalBiochem CP06, 1:10,000 dilution for WB)
Pol kappa [A-9] (Santa Cruz Biotechnology sc-166667, 1:1000 dilution for WB)
Flag [M2] (Sigma-Aldrich F1804, 1:5000 dilution for WB)
53BP1 (Abcam ab175933, 1:200 dilution for IF)
Cyclin A2 (Calbiochem CC17, 1:100 dilution for IF)
Goat anti-mouse IgG (H+L) Alexa Fluor 488 (Thermo Fisher A11001, 1:350 dilution for DNA fiber assay)
Goat anti-rat IgG (H+L) Alexa Fluor 594 (Thermo Fisher A11007, 1:350 dilution for DNA fiber assay)
Goat anti-mouse IgG (H+L) Alexa Fluor 546 (Thermo Fisher A11003, 1:350 dilution for IF)
Goat anti-rabbit IgG Alexa Fluor 488 (Thermo Fisher A11008, 1:350 dilution for IF)
Peroxidase AffiniPure Goat Anti-Mouse IgG (H+L) (Jackson Labs 115-035-003, 1:10,000 dilution for WB)
Peroxidase AffiniPure Goat Anti-Rabbit IgG (H+L) (Jackson Labs 111-035-003, 1:10,000 dilution for WB)

Validation

https://www.bdbiosciences.com/en-us/products/reagents/flow-cytometry-reagents/clinical-discovery-research/single-color-antibodies-ruo-gmp/purified-mouse-anti-brdu.347580
https://www.abcam.com/brdu-antibody-bu175-icr1-proliferation-marker-ab6326.html
https://www.bethyl.com/product/A301-700A/USP1+Antibody
https://www.bethyl.com/product/A300-191A?referrer=search
https://www.abcam.com/pcna-antibody-pc10-ab29.html
https://www.abcam.com/chk1-antibody-e250-ab32531.html
https://www.cellsignal.com/products/primary-antibodies/ubiquityl-pcna-lys164-d5c7p-rabbit-mab/13439
https://www.cellsignal.com/products/primary-antibodies/phospho-chk1-ser345-133d3-rabbit-mab/2348?site-search-type=Products&N=4294956287&Ntt=ser345+chk1&fromPage=plp
https://www.emdmillipore.com/US/en/product/Anti-Tubulin-Mouse-mAb-DM1A,EMD_BIO-CP06?ReferrerURL=https%3A%2F%2Fwww.google.com%2F
https://www.scbt.com/p/dinb-antibody-a-9
https://www.sigmaaldrich.com/US/en/product/sigma/f3165?gclid=CjwKCAjw9aiIBhA1EiwAJ_GTSplthjNIGWkhq5fe2AFowOZt4mEj-W9gHyuiZ4F2q4qWCWoyvO2YzhoCPJAQAvD_BwE
https://www.abcam.com/53bp1-antibody-ab21083.html
https://www.thermofisher.com/antibody/product/Cyclin-A2-Antibody-clone-E23-1-Monoclonal/MA1-154

Eukaryotic cell lines

Policy information about cell lines

Cell line source(s)

HCT116, U2OS, HEK293T cell lines were obtained from ATCC.

Authentication

no cell line authentication was performed

Mycoplasma contamination	All cell lines were tested for mycoplasma using the ROche MycoTOOL detection. Mycoplasma contamination was not detected.
Commonly misidentified lines (See ICLAC register)	no commonly misidentified lines were used
